# A systematic review and Bayesian network meta-analysis comparing left bundle branch pacing, his bundle branch pacing, and right ventricular pacing for atrioventricular block

**DOI:** 10.3389/fcvm.2022.939850

**Published:** 2022-10-25

**Authors:** Yue Zhang, Yuan Jia, Jia Liu, Rongpin Du

**Affiliations:** ^1^Graduate School of Hebei Medical University, Shijiazhuang, China; ^2^Department of Cardiology, Hebei General Hospital, Shijiazhuang, China

**Keywords:** network meta-analysis, left bundle branch pacing, left bundle branch area pacing, His–Purkinje system pacing, atrioventricular block

## Abstract

**Background:**

Although right ventricular pacing (RVP) is recommended by most of the guidelines for atrioventricular block, it can cause electrical and mechanical desynchrony, impair left ventricular function, and increase the risk of atrial fibrillation. Recently, the His–Purkinje system pacing, including His bundle pacing (HBP) and left bundle branch pacing (LBBP), has emerged as a physiological pacing modality. However, few studies have compared their efficacy and safety in atrioventricular block (AVB).

**Methods and results:**

The PubMed, Web of Science, Cochrane Library, and ScienceDirect databases were searched for observational studies and randomized trials of patients with atrioventricular block requiring permanent pacing, from database inception until 10 January 2022. The primary outcomes were complications and heart failure hospitalization. The secondary outcomes included changes in left ventricular ejection fraction (LVEF) and left ventricular end-diastolic diameter (LVEDD), pacing parameters, procedure duration, and success rate. After extracting the data at baseline and the longest follow-up duration available, a pairwise meta-analysis and a Bayesian random-effects network meta-analysis were performed. Odds ratios (ORs) with 95% confidence intervals (CIs) or 95% credible intervals (CrIs) were calculated for dichotomous outcomes, whereas mean differences (MDs) with 95% CIs or 95% CrIs were calculated for continuous outcomes. Seven studies and 1,069 patients were included. Overall, 43.4% underwent LBBP, 33.5% HBP, and 23.1% RVP. Compared with RVP, LBBP and HBP were associated with a shorter paced QRS duration and a more preserved LVEF. HBP significantly increased the pacing threshold and reduced the R-wave amplitude. There was no difference in the risk of complications or the implant success rate. The pacing threshold remained stable during follow-up for the three pacing modalities. The pacing impedance was significantly reduced in HBP, while a numerical but non-significant pacing impedance decrease was observed in both LBBP and RVP. LBBP was associated with an increased R-wave amplitude during follow-up.

**Conclusion:**

In this systematic review and network meta-analysis, HBP and LBBP were superior to RVP in paced QRS duration and preservation of LVEF for patients with atrioventricular block. LBBP was associated with a lower pacing threshold and a greater R-wave amplitude than HBP. However, the stability of the pacing output of LBBP may be a concern. Further investigation of the long-term efficacy in left ventricular function and the risk of heart failure hospitalization is needed.

**Systematic review registration:**

[https://www.crd.york.ac.uk/prospero/display_record.php?RecordID=315046], identifier [CRD42022315046].

## Introduction

Right ventricular pacing (RVP) is the traditional pacing modality recommended for patients with atrioventricular block by most of the guidelines (1, 2), with a shorter procedure time and an easier learning curve. However, RVP with a high ventricular pacing rate can increase the risk of atrial fibrillation, pacing-induced cardiomyopathy, heart failure hospitalization, and death (3, 4).

Recent scientific evidence has shown the efficacy and safety of the His–Purkinje system pacing, with significant improvements in exercise capacity, ventricular synchrony, left ventricular ejection fraction (LVEF), and so on (5). Few studies have compared the effectiveness of left bundle branch pacing (LBBP), His bundle pacing (HBP), and RVP in patients with atrioventricular block, especially LBBP vs. HBP. Thus, we aimed to comprehensively compare the clinical outcomes and pacing parameters of these three pacing modalities for atrioventricular block.

The evidence was assessed in a network meta-analysis. Network meta-analyses synthesize direct and indirect evidence in a network of trials that compare multiple interventions (6). This method allows for a comparison of the three pacing modalities for atrioventricular block despite the paucity of head-to-head comparisons.

## Methods

This is a systematic review and network meta-analysis of pacing modality intervention trials in atrioventricular block. The research question was developed with the PICOS framework as follows:

Participants: Patients with atrioventricular block.

Intervention and comparator: Left bundle branch pacing, His bundle pacing, and right ventricular pacing.

Outcomes: (1) Pacing parameters, including paced QRS duration (ms), pacing impedance (Ω), pacing threshold (V), and R-wave amplitudes (mV). (2) Clinical outcomes, including complications and heart failure hospitalization. (3) Left ventricular function, including LVEF (%) and left ventricular end-diastolic diameter (LVEDD) (mm). (4) Procedure duration (min) and success rate.

Studies: Observational studies and randomized trials.

Reporting was conducted according to the Preferred Reporting Items for Systematic Review and Network Meta-analysis (PRISMA-NMA) statement (7). This study was registered at the Prospective International Register of Systematic Reviews (PROSPERO). The registration number is CRD42022315046.

### Data sources

The PubMed, Web of Science, Cochrane Library, and ScienceDirect databases were consulted to identify English-language studies on LBBP, HBP, and RVP for the treatment of atrioventricular block from database inception until 10 January 2022. Details of the electronic search strategies are summarized in the Supplementary materials.

### Study selection criteria

Eligible studies included observational studies and randomized trials comparing the effects of LBBP or HBP vs. RVP for atrioventricular block in pacing parameters, clinical outcomes, left ventricular function, procedure duration, and success rate. Exclusion criteria were studies with population or outcome stratification not of interest, or with fewer than 10 patients per study group. No additional information was requested from the study authors.

### Study identification

Two investigators (YZ and YJ) individually screened the articles by title, abstract, and full text. The inclusion of a study was decided by consensus between the two investigators. Disagreements were resolved by discussion, and if no agreement could be reached, a third senior investigator (JL) made the decision.

### Outcomes and data extraction

The primary outcomes were complications and heart failure hospitalization. The secondary outcomes included changes in LVEF and LVEDD, pacing parameters, procedure duration, and success rate. The pacing threshold was the His lead for HBP and LBBP and RV lead for RVP at 0.4, 0.5, or 1.0 ms. The complications included those requiring treatment or reintervention during the perioperative period or at follow-up. Supplementary Table 1 shows the detailed definitions of the complications reported by the included studies.

For each outcome, data at baseline and the longest available follow-up time point were extracted. Other extracted data included characteristics of the study design, baseline demographic characteristics (age, sex, number of patients), duration of treatment, and follow-up duration.

For the randomized crossover trials, data were included from the first period, before crossing over, to avoid the risk of any carryover effect (8).

### Risk of bias and publication bias assessment

The quality of the included studies was assessed using the Newcastle–Ottawa Scale (NOS) for observational studies and the risk of bias 2 tool (ROB 2.0) for randomized trials. Publication bias was assessed with funnel plots and Egger’s test for every outcome comparison.

### Data analysis

The initial analysis consisted of a two-group outcome comparison between LBBP or HBP and RVP for all outcomes. Then, for each endpoint, a Bayesian random-effects NMA was conducted with the three pacing strategies. Odds ratios (ORs) with 95% confidence intervals (CIs) or 95% credible intervals (CrIs) were calculated for dichotomous outcomes, whereas mean differences (MDs) with 95% CIs or 95% CrIs were calculated for continuous outcomes. The *I*^2^ index was calculated to assess heterogeneity. An *I*^2^ of less than 25% was viewed as low heterogeneity, between 25% and 50% as moderate, and over 50% as high heterogeneity (9). Treatments for each outcome were ranked based on the surface under the cumulative ranking curve (SUCRA) method, which vary from 0 to 100% and represent the probability that the treatment evaluated is the best. All analyses were conducted using RevMan version 5.4.1, R version 4.1.2 with the “gemtc” and “netmeta” packages, and JAGS 4.3.0.

## Results

**FIGURE 1:**
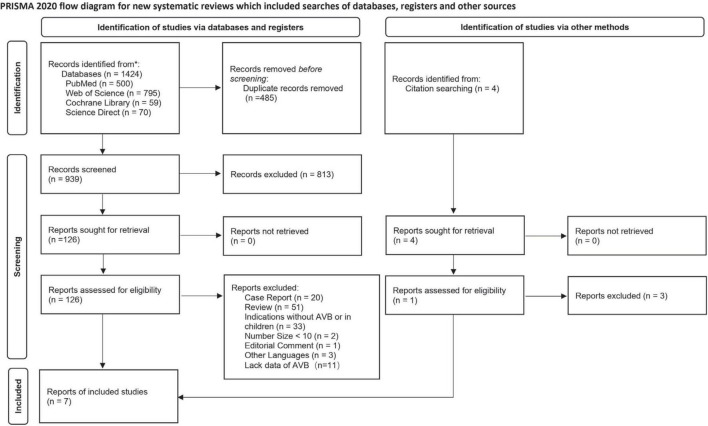
Flowchart of the literature selection process.

In total, 1,428 studies were retrieved, of which 485 duplicates were excluded. A total of 813 irrelevant records were excluded by a screening of titles and abstracts. After a full-text assessment of the remaining 130 articles, 7 studies met the pre-defined inclusion criteria and were included in the meta-analysis (10–16). The flowchart of the literature selection process is shown in Figure 1.

Among the seven included studies, three compared LBBP with RVP (*n* = 339 vs. *n* = 216 patients), three compared LBBP with HBP (*n* = 228 vs. *n* = 260 patients), and 1 compared HBP with RVP (*n* = 19 vs. *n* = 19 patients) with follow-up durations between 3 and 24 months. Initial enrollment ranged from 2007 to 2020. Five were observational studies, one was a randomized controlled trial, and one was a randomized crossover trial. In total, 1,069 patients from 11 centers across five countries were included. The pacing indication was atrioventricular block, and 736 (68.8%) patients in six studies had a preserved left ventricular ejection fraction > 40%. The mean age of the patient population was 67.7 years. Of these, 464 (43.4%) underwent LBBP, 247 (23.1%) HBP, and 358 (33.5%) RVP. The overall success rate of LBBP was 93.5% (360/385). The characteristics of the included trials are presented in Table 1.

**TABLE 1:** Demographic data of all studies.

Author, year	Study design	Country	Indication	Follow up (months)	Male, *n*	Pacing mode	Number of patients, *n*	Success rate (%)	Baseline LVEF (%)	3830 used
Hu et al. (10)	Observational	China	AVB	3	32	HBP	25	76	59.3 ± 11.3	Yes
						LBBP	25	88	57.7 ± 7.8	Yes
Hasumi et al. (13)	Observational	Japan	AVB with preserved LVEF	6	NR	HBP	21	64	NR	Yes
						LBBP	71	81	NR	Yes
Vijayaraman et al. (15)	Observational	USA	Advanced AVB	12∼24	211	HBP	182	NR	NR	Yes
						LBBP	151	NR	NR	Yes
Li et al. (11)	Observational	China	AVB and LVEF > 50% at baseline	12	242	RVP	246	100	61.5 ± 6.4	–
						LBBP	120	95.5	61.7 ± 7.4	Yes
Zhang et al. (14)	Observational	China	AVB	12∼24	30	RVP	33	100	56.29 ± 5.40	–
						LBBP	37	87.9	55.08 ± 4.32	Yes
Riano Ondiviela et al. (12)	Randomized controlled trial	Spain	Third-degree AVB with preserved LVEF	3	37	RVP	60	95	NR	NR
						LBBP	60	95	NR	
Kronborg et al. (16)	Randomized crossover trial	Denmark	AVB with a preserved LVEF > 40%	12	30	RVP	19	97	NR	–
						HBP	19	84	NR	Yes

AVB, atrioventricular block; HBP, His bundle pacing; LBBP, left bundle branch pacing; LVEF, left ventricular ejection fraction; NR, not reported; RVP, right ventricular pacing.

### Risk of bias for individual studies

The NOS for observational studies ranged from 3 to 8, the ROB 2.0 for the randomized crossover trial was low risk, and for the randomized controlled trial, there were some concerns (see Supplementary Table 2 and Supplementary Figure 1).

#### Pairwise meta-analysis

Among the seven included studies, three compared LBBP with RVP, three compared LBBP with HBP, and one compared HBP with RVP. The pairwise meta-analysis could only be conducted for LBBP vs. RVP and LBBP vs. HBP. Supplementary Figure 2 shows the outcomes and studies included in the pairwise meta-analyses. The results of the pairwise meta-analysis are summarized in Table 2. Compared with HBP, LBBP was associated with a lower pacing threshold, greater R-wave amplitude, and higher pacing impedance at follow-up, while there was no significant difference in procedure duration, paced QRS duration, pacing impedance after implantation, or risks of complications. LBBP demonstrated significant improvements over RVP in terms of a shorter paced QRS duration, more preserved LVEF, smaller LVEDD, and reduced risk of heart failure hospitalization. LBBP was associated with lower pacing impedance after implantation than RVP, with no difference in pacing impedance at follow-up, implant success rate, pacing threshold, R-wave amplitudes, or complications. Supplementary Figures 4–26 show the forest plots for the corresponding outcomes.

**TABLE 2:** Results of pairwise meta-analyses.

		Pairwise meta-analysis			

		Procedure duration (min)		Implant success rate	
		*N, n*	OR; 95%CI; *p*	*N, n*	OR; 95%CI; *p*
LBBP vs. HBP		2 (207 vs. 168)	–0.57; (–21.24, 20.11); 0.96	NA	
LBBP vs. RVP		NA		3 (339 vs. 217)	0.27; (0.04,1.80); 0.18
HBP vs. RVP		NA		NA	

		**Pacing parameters**			

		**QRS duration (ms)**		**Pacing threshold (V)**	
		***N, n***	**MD; 95%CI; *p***	***N, n***	**MD; 95%CI; *p***

LBBP vs. HBP	Baseline	2 (207 vs. 168)	11.74; (–5.76, 29.24); 0.19	2 (207 vs. 168)	**–0.58; (–0.69, –0.47); <0.00001**
	Follow-up	2 (207 vs. 168)	–3.32; (–9.57, 2.93); 0.3	2 (207 vs. 168)	**–0.59; (–0.72, –0.46); <0.00001**
LBBP vs. RVP	Baseline	3 (339 vs. 217)	2.20; (–3.11, 7.51); 0.42	3 (339 vs. 217)	0.02; (–0.05, 0.08); 0.61
	Follow-up	3 (339 vs. 217)	**–42.43; (–44.68, –40.18); <0.00001**	2 (279 vs. 157)	0.01; (–0.13, 0.14); 0.94
HBP vs. RVP	Baseline	NA		NA	
	Follow-up				

		**Pacing parameters**			

		***R* wave amplitude (mV)**		**Pacing impedance (**Ω**)**	
		***N, n***	**MD; 95%CI; *p***	***N, n***	**MD; 95%CI; *p***

LBBP vs. HBP	Baseline	2 (207 vs. 168)	**7.95; (7.01, 8.89); <0.00001**	2 (207 vs. 168)	107.71; (-101.90, 317.32); 0.31
	Follow-up	2 (207 vs. 168)	**9.73; (4.64, 14.82); 0.0002**	2 (207 vs. 168)	**36.69; (22.51, 50.86); <0.00001**
LBBP vs. RVP	Baseline	3 (339 vs. 217)	0.73; (–1.23, 2.70); 0.46	2 (279 vs. 157)	**–68.48; (–136.40, –0.55); 0.05**
	Follow-up	2 (279 vs. 157)	0.85; (–1.03, 2.72); 0.38	2 (279 vs. 157)	–94.96; (–211.65, 21.73); 0.11
HBP vs. RVP	Baseline	NA		NA	
	Follow-up				

		**Left ventricular function**			

		**LVEF (%)**		**LVEDD (mm)**	
		***N, n***	**MD; 95%CI; p**	***N, n***	**MD; 95%CI; *p***

LBBP vs. HBP	Baseline	NA		NA	
	Follow-up				
LBBP vs. RVP	Baseline	2 (279 vs. 157)	–0.22; (–1.49, 1.04); 0.73	2 (279 vs. 157)	0.67; (–1.27, 2.60); 0.50
	Follow-up	2 (279 vs. 157)	**4.32; (3.02, 5.61); <0.00001**	2 (279 vs. 157)	**–3.63; (–6.46, –0.80); 0.01**
HBP vs. RVP	Baseline	NA		NA	
	Follow-up				

		**Clinical outcomes**			

		**Complications**		**Heart failure hospitalization**	
		***N, n***	**OR; 95%CI; *p***	***N, n***	**OR; 95%CI; *p***

LBBP vs. HBP		2 (207 vs. 168)	1.30; (0.57, 2.97); 0.53	NA	
LBBP vs. RVP		3 (339 vs. 217)	0.77; (0.24, 2.46); 0.66	2 (279 vs. 157)	**0.21; (0.08, 0.53); 0.001**
HBP vs. RVP		NA		NA	

		**Chronic evolution of pacing parameters**			

		**Pacing threshold (V)**		**Pacing impedance (Ω)**	
	***N, n***	**MD; 95%CI; *p***	***N, n***	**MD; 95%CI; *p***

LBBP	4 (486)	–0.04; (–0.13, 0.05); 0.37	4 (486)	103.38; (–21.01, 227.77); 0.1
HBP	3 (206)	–0.08; (–0.23, 0.08); 0.34	3 (206)	**71.04; (24.24, 117.83); 0.003**
RVP	3 (195)	–0.01; (–0.04, 0.02); 0.48	3 (195)	76.74; (–6.18, 159.67); 0.07

	**R wave amplitude (mV)**			

	***N, n***	**MD; 95%CI; *p***		
LBBP	4 (486)	**–2.12; (–4.05, –0.20); 0.03**		
HBP	3 (206)	–0.10; (–0.66, 0.45); 0.71		
RVP	3 (195)	–0.89; (–3.36, 1.58); 0.48		

Bold values indicate statistical differences. CI, confidence interval; HBP, His bundle pacing; LBBP, left bundle branch pacing; LVEF, left ventricular ejection fraction; MD, mean difference; N, number of studies; n, number of participants; NA, not applicable; RVP, right ventricular pacing.

##### Pacing parameters

###### Paced QRS duration

Left bundle branch pacing was associated with a significantly shorter paced QRS duration than RVP (MD, –42.42; 95% CI, –44.68 to –40.17; *p* < 0.00001; *I*^2^ = 18%). LBBP did not shorten the paced QRS duration relative to HBP (MD, –3.32; 95% CI, –9.57 to 2.93; *p* = 0.30; *I*^2^ = 41%).

###### Pacing impedance

Compared with RVP, LBBP demonstrated a lower pacing impedance at the time of implantation (MD, –68.48; 95% CI, –136.40 to –0.55; *p* = 0.05; *I*^2^ = 52%), with no significant difference at follow-up (MD, –94.96; 95% CI, –211.65 to 21.73; *p* = 0.11; *I*^2^ = 87%). There was no significant difference in pacing impedance between LBBP and HBP after implantation (MD, 107.71; 95% CI, –101.90 to 317.32; *p* = 0.31; *I*^2^ = 96%), but LBBP resulted in a significantly higher pacing impedance compared with HBP at follow-up (MD, 36.69; 95% CI, 22.51 to 50.86; *p* < 0.00001; *I*^2^ = 0%).

###### Pacing threshold

The pacing threshold in the LBBP group was significantly lower than in the HBP group at the time of implantation (MD, –0.58; 95% CI, –0.69 to –0.47; *p* < 0.00001; *I*^2^ = 0%) and follow-up (MD, –0.59; 95% CI, –0.72 to –0.46; *p* < 0.00001; *I*^2^ = 0%). There was no significant difference between LBBP and RVP in the pacing threshold, whether at the time of implantation or follow-up.

###### R-wave amplitude

Left bundle branch pacing was associated with a higher R-wave amplitude than HBP at the time of implantation (MD, 7.95; 95% CI, 7.01 to 8.89; *p* < 0.00001; *I*^2^ = 0%) and follow-up (MD, 9.73; 95% CI, 4.64 to 14.82; *p* = 0.0002; *I*^2^ = 94%). There was no significant difference between LBBP and RVP at the time of implantation or follow-up.

##### Left ventricular function

There was only one included study (10) comparing LBBP and HBP reporting LVEF and LVEDD. Hu et al. (10) found that there was no statistical difference in LVEF (*p* = 0.764) or LVEDD (*p* = 0.957) at the 3-month follow-up between LBBP and HBP.

###### Left ventricular ejection fraction

No significant difference was found in baseline LVEF between LBBP and RVP. At follow-up, LBBP demonstrated a higher LVEF than RVP (MD, 4.32; 95% CI, 3.02 to 5.61; *p* < 0.00001; *I*^2^ = 0%).

###### Left ventricular end-diastolic diameter

No statistically significant difference was found in baseline LVEDD between LBBP and RVP. At follow-up, LBBP was associated with a smaller LVEDD than RVP (MD, –3.63; 95% CI, –6.46 to –0.80; *p* = 0.01; *I*^2^ = 88%).

##### Clinical outcomes

###### Complications

There was no significant difference in the risk of complications, whether between LBBP and RVP or between LBBP and HBP.

###### Heart failure hospitalization

Left bundle branch pacing reduced the risks of heart failure hospitalization in comparison with RVP (MD, 0.21; 95% CI, 0.08 to 0.53; *p* = 0.001; *I*^2^ = 0%). None of the included studies compared the risk of heart failure hospitalization between LBBP and HBP.

#### Chronic evolution of pacing parameters

To explore the stability of the pacing output for the three pacing modalities, a pairwise meta-analysis was conducted to compare the changes in pacing parameters during follow-up. Supplementary Figures 27–35 show the corresponding forest plots.

For LBBP, the R-wave amplitude increased during follow-up (MD, –2.12; 95% CI, –4.05 to –0.20; *p* = 0.03; *I*^2^ = 86%), while the pacing threshold remained stable. A numerical but non-significant decrease in the pacing impedance was observed in both LBBP and RVP. The pacing threshold and R-wave amplitude remained stable during follow-up in the RVP group. HBP demonstrated a decreased pacin gimpedance at follow-up (MD, 71.04; 95% CI, 24.24–117.83; *p* = 0.003; *I*^2^ = 79%), while pacing threshold and R-wave amplitude remained stable.

#### Network meta-analysis

Supplementary Figure 3 shows the studies and selected outcomes included in the network meta-analyses. Both LBBP and HBP shortened the paced QRS duration and improved LVEF compared with RVP. HBP increased the pacing threshold after implantation and at follow-up, and reduced the R-wave amplitude after implantation. Network meta-analysis showed that there was no difference in success rate, complications, or pacing impedance after implantation or at follow-up among the three pacing modalities. Indirect comparisons showed that there was no difference in procedure duration or LVEDD at follow-up. Table 3 shows the league tables for procedure duration, implant success, pacing parameters, left ventricular function, and clinical outcomes. The network plots for all the outcomes are shown in Supplementary Figures 36, 37.

**TABLE 3:** League tables of network meta-analysis.

*Procedure duration (min)			Success rate		
LBBP			LBBP		
–0.63 (–24.64, 23.54)	HBP		0.56 (–2.54, 3.36)	HBP	
24.26 (–10.48, 59.21)	24.86 (–17.93, 66.58)	RVP	–2.06 (–5.04, –0.04)	–2.65 (–5.90, 0.20)	RVP

**Baseline QRS duration (ms)**			**Paced QRS duration (ms)**		

LBBP			LBBP		
10.12 (–4.30, 21.92)	HBP		–2.45 (–8.84, 3.16)	HBP	
3.86 (–6.61, 14.38)	–6.19 (–19.15, 9.23)	RVP	–**42.75 (**–**47.75,** –**38.60)**	–**40.33 (**–**46.67,** –**33.74)**	RVP

**Baseline pacing impedance (**Ω**)**			**Pacing impedance at follow-up (**Ω**)**		

LBBP			LBBP		
95.56 (–68.86, 253.21)	HBP		2.89 (–112.49, 121.58)	HBP	
–139.22 (–323.81, 48.50)	–44.05 (–204.39, 118.86)	RVP	–67.35 (–183.21, 49.64)	–70.57 (–203.51, 62.04)	RVP

**Baseline pacing threshold (V)**			**Pacing threshold at follow-up (V)**		

LBBP			LBBP		
–**0.67 (**–**1.10,** –**0.35)**	HBP		–**0.73 (**–**1.40,** –**0.12)**	HBP	
0.06 (–0.18, 0.42)	**0.73 (0.41, 1.27)**	RVP	0.14 (–0.45, 0.82)	**0.88 (0.19, 1.70)**	RVP

**Baseline R wave amplitude (mV)**			**R wave amplitude at follow-up (mV)**		

LBBP			LBBP		
**7.29 (4.25, 10.21)**	HBP		**8.43 (1.32, 15.27)**	HBP	
0.92 (–1.55, 3.48)	–**6.36 (**–**9.56,** –**2.89)**	RVP	2.13 (–4.84, 9.15)	–6.28 (–14.21, 1.76)	RVP

*Baseline LVEF (%)			**LVEF at follow-up (%)**		

LBBP			LBBP		
–1.03 (–6.26, 4.75)	HBP		–0.57 (–5.16, 3.96)	HBP	
–0.31 (–2.22, 1.46)	0.66 (–5.32, 6.34)	RVP	**4.33 (1.43, 7.32)**	**4.91 (0.53, 9.44)**	RVP

*Baseline LVEDD (mm)			*LVEDD at follow-up (mm)		

LBBP			LBBP		
–0.55 (–5.58, 4.37)	HBP		–0.19 (–7.03, 6.87)	HBP	
0.57 (–1.30, 2.59)	1.13 (–4.17, 6.57)	RVP	–3.61 (–8.21, 0.88)	–3.45 (–11.72, 4.65)	RVP

**Complications**					

LBBP					
0.36 (–0.77, 1.64)	HBP				
–0.18 (–1.56, 1.30)	–0.55 (–2.35, 1.29)	RVP			

Bold values indicate statistical differences. *Comparisons between HBP and RVP were indirect. HBP, His bundle pacing; LBBP, left bundle branch pacing; LVEF, left ventricular ejection fraction; LVEDD, left ventricular end-diastolic diameter; LV function, left ventricular function; RVP, right ventricular pacing.

##### Procedure duration

No significant difference was observed in procedure duration for any comparisons. The comparison between HBP and RVP was indirect.

##### Implant success

None of the comparisons showed significant differences in implant success.

##### Paced QRS duration

There was no significant difference in baseline QRS duration among the three groups. However, at follow-up, the paced QRS duration of RVP was significantly higher than that of LBBP (MD, 42.75; 95% CrI, 38.60 to 47.75) and HBP (MD, 40.33; 95% CrI, 33.74 to 46.67), while there was no significant difference between LBBP and HBP.

##### Pacing impedance

No significant difference was observed for any comparisons in pacing impedance, whether after implantation or during follow-up.

##### Pacing threshold

In the NMA, HBP increased the pacing threshold compared with LBBP (MD, 0.67; 95% CrI, 0.35 to 1.10) and RVP (MD, 0.73; 95% CrI, 0.41 to 1.27) after implantation. At follow-up, HBP increased the pacing threshold compared with LBBP (MD, 0.73; 95% CrI, 0.12 to 1.40) and RVP (MD, 0.88; 95% CrI, 0.19 to 1.70). No significant difference was observed for LBBP vs. RVP.

##### R-wave amplitude

His bundle pacing exerted a lower R-wave amplitude compared with LBBP (MD, –7.29; 95% CrI, –10.21 to –4.25) and RVP (MD, –6.36; 95% CrI, –9.56 to –2.89) after implantation. HBP decreased the R-wave amplitude relative to LBBP at follow-up (MD, –8.43; 95% CrI, –15.27 to –1.32). No significant difference was observed for LBBP vs. RVP.

##### Left ventricular function assessment

###### Left ventricular ejection fraction

There was no significant difference in LVEF at admission among the three groups. At follow-up, RVP decreased LVEF relative to HBP (MD, –4.91; 95% CrI, –9.44 to –0.53) and LBBP (MD, –4.33; 95% CrI, –7.32 to –1.43). There was no significant difference between LBBP and HBP.

###### Left ventricular end-diastolic diameter

Additionally, there was no significant difference in LVEDD at baseline or follow-up. The comparison between HBP and RVP was indirect.

##### Complications

No major differences among the three pacing modalities were observed.

##### Subgroup analysis

Subgroup analyses were performed by excluding the randomized trials. Supplementary Table 3 shows that the subgroup analyses were consistent with the main analysis except for the results of implant success rate and LVEF at follow-up, which may be explained by the small number of studies and indirect comparisons between HBP and RVP after excluding the randomized trials.

#### Ranking results

The SUCRA ranking results (Table 4) showed that RVP had the highest probability of being the best intervention for a shorter procedure duration, higher implant success rate, greater pacing impedance, and lower pacing threshold based on the SUCRA value (91.3%, 97.1%, 84.1%/88.4%, and 88.4%/88.2%, respectively). HBP was ranked the top one for fewer complications (72.9%) and more preserved LVEF at follow-up (79.3%). LBBP was the top one in terms of a shorter paced QRS duration (91.9%), higher R-wave amplitude after implantation (91.4%) and at follow-up (88.8%), and smaller LVEDD at follow-up (74.0%). However, considering that the sample sizes of the different interventions varied greatly, the results might be highly biased and should be interpreted with caution.

**TABLE 4:** Relative rankings of HBP, LBBP, and RVP based on SUCRA values (*after implantation/at follow-up).

Outcomes	Procedure duration (min)	Implant success	Paced QRS duration (follow-up) (ms)	Pacing impedance* (Ω)	Pacing threshold* (V)	R-wave amplitude* (mV)	LVEF (follow-up) (%)	LVEDD (follow-up) (mm)	Complications
	
Pacing modality									
	SUCRA (%)	SUCRA (%)	SUCRA (%)	SUCRA (%)	SUCRA (%)	SUCRA (%)	SUCRA (%)	SUCRA (%)	SUCRA (%)
LBBP	29.9	34.5	**91.9**	57.9/31.5	61.2/60.5	**91.4/88.8**	69.5	**74.0**	43.3
HBP	28.8	18.4	58.1	8.1/30.1	0.4/1.3	0.2/3.1	**79.3**	64.7	**72.9**
RVP	**91.3**	**97.1**	0.0	**84.1/88.4**	**88.4/88.2**	58.4/58.1	1.13	11.4	33.9

HBP, His bundle pacing; LBBP, left bundle branch pacing; LVEF, left ventricular ejection fraction; LVEDD, left ventricular end-diastolic diameter; RVP, right ventricular pacing; SUCRA, surface under the cumulative ranking curve. Bold values are the top one value of SUCRA ranking.

#### Sensitivity analysis

Sensitivity analysis was performed by comparing the results of network meta-analysis between the Bayesian framework and the frequentist framework (Supplementary Table 5). Overall, the sensitivity analysis was consistent with the main analysis except for the procedure duration, implant success rate, R-wave amplitude, and LVEDD at follow-up, which may be restricted by the small sample size.

#### Publication of bias assessment

Supplementary Figures 38–40 show the funnel plots and results of Egger’s test for every outcome comparison.

## Discussion

After combining the direct and indirect evidence, we obtained several important findings: (a) Compared with RVP, LBBP and HBP were associated with a shorter paced QRS duration and more preserved LVEF. (b) HBP significantly increased the pacing threshold and reduced the R-wave amplitude. (c) There was no difference in the risk of complications and implant success rate. However, some debatable results need further discussion, (a) LBBP demonstrated a higher pacing impedance at follow-up than HBP and a lower pacing impedance after implantation than RVP in pairwise meta-analysis. Further analysis showed that during follow-up, there was a significant impedance decrease in the HBP group, while a numerical impedance decrease was observed in LBBP and RVP. Pacing impedance may decrease when a lead insulation breach or intracavity lead dislodgement occurs (17). For LBBP, lead dislocation was the most common complication (10 in 427, 2.3%) as shown in Supplementary Table 1. However, the lead dislodgement rate of HBP is relatively low. In the included 228 HBP cases, only one patient developed lead dislodgement. Besides, some pathophysiological changes, such as pneumothorax and pericardial or pleural effusion, can cause indefinite impedance changes. Supplementary Table 1 shows that two patients developed pneumothorax, and two suffered from pericardial effusion in the HBP group. In addition, the possibility of local fibrosis cannot be excluded. However, whether these conditions are the determinants of the impedance change remains unknown. Besides, due to the small number of included studies, there was a high heterogeneity, so further investigation is needed. (b) Network meta-analysis showed that there was no difference in LVEDD among the three pacing modalities, while pairwise meta-analysis showed that LBBP could reduce LVEDD compared with RVP. SUCRA ranking results also showed that LBBP was the top one for a smaller LVEDD at follow-up. The possible reasons behind this inconsistency may be bias caused by the small number of included studies and indirect comparisons between HBP and RVP. (c) Only two included studies compared LBBP versus RVP reported the rates of heart failure hospitalization (HFH). Comparisons of LBBP vs. HBP and HBP vs. RVP were missing, so the risk of HFH remains unknown for these procedures.

Permanent pacemaker implantation is a common approach to the management of bradycardia and conduction system disease. RVP has been the standard therapy with easy implantation and stable long-term pacing parameters. The current 2018 multi-society guideline on the evaluation and management of patients with bradycardia and cardiac conduction delay lists RVP as the only recommended pacing strategy for patients with EF more than 50% (class IIa) (1). The 2021 ESC guideline on cardiac pacing and cardiac resynchronization therapy suggests that HBP may be considered as an alternative to RV pacing in patients with AVB and LVEF > 40%, who are anticipated to have > 20% ventricular pacing (class IIb) (2). Long-term RVP can promote fibrosis and disarrays of endocardial myocytes and myofibrils (18, 19), cause asynchronous ventricular contraction, and negatively affect the hemodynamic status, leading to pacemaker-induced cardiomyopathy (PCM) and a deterioration of heart function (20, 21). PCM is defined as a drop in the left ventricular ejection fraction (LVEF) of more than 10% from baseline after excluding other differential diagnoses (4, 20). It has been reported that the prolongation of paced QRS duration, as a surrogate marker of interventricular desynchrony, has a significant correlation with PCM (22). Our study supported that physiologic pacing, both HBP and LBBP, is associated with a narrower paced QRS duration compared to RVP, which may confer a lower risk of developing pacing-induced cardiomyopathy. Additional studies are required to determine whether LBBP or HBP could be the first-line approach for pacing.

For paced QRS duration and LVEF at follow-up, there was no significant difference between LBBP and HBP in the meta-analysis. Theoretically, HBP was more physiologic than LBBP, which may lead to a shorter paced QRS duration in the HBP group. However, SUCRA ranking results showed that LBBP was the top one for a shorter paced QRS duration. First, the non-selective HBP produces a longer paced QRS duration compared with selective HBP, which may affect the overall paced QRS duration in the HBP arm. Second, the rapid conduction velocity in the Purkinje fibers may result in fast retrograde activation of the right bundle, leading to a short paced QRS duration in LBBP. In our analysis, although LBBP (SUCRA 91.9%) is slightly more advantageous than HBP (SUCRA 58.1%) in a shorter paced QRS duration, SUCRA ranking results showed that HBP was ranked the top one for a more preserved LVEF at follow-up. There may be several possible reasons. While it has been shown that a narrower QRS in biventricular stimulation implies better clinical results, it has not been confirmed that this is also true regarding the conduction system pacing. Ultra-high-frequency electrocardiography (UHF-ECG) is another tool for ventricular dyssynchrony assessment. Studies by Curila et al. showed that there was no difference in the electrical ventricular synchrony measured by UHF-ECG between selective and non-selective HBP, although the paced QRS durations differ (23). In another study, Curila et al. reported that LBBP caused less physiological ventricular depolarization compared to HBP using UHF-ECG (24), which may affect further left ventricular function. Besides, there may be other factors related to left ventricular function. The paced QRS axis, which may be a predictor of pacing-induced left ventricular dysfunction (25), remained identical to the intrinsic one no matter in the selective or the non-selective HBP (26). However, Hu et al. (10) observed a 40.9% (9 in 22) left axis deviation of paced QRS in the LBBP group, higher than those in HBP. Moreover, HBP can also reduce T peak to T end (Tp-Te) duration, which is associated with arrhythmia and mortality (27). Whether LBBP may change the Tp-Te duration or not is unknown. We need more trials to evaluate the difference in these metrics between LBBP and HBP, and whether these will affect left ventricular function. A network meta-analysis in patients requiring cardiac resynchronization therapy (CRT) (28) reported that LBBP (SUCRA 97.2%) was the best treatment for improvements of LVEF, followed by HBP (SUCRA 52.5%). This may be explained by the difference in the pacing indications and baseline LVEF. Most of the CRT patients in the meta-analysis conducted by Juan Hua et al. (28) had a baseline LVEF < 35%, while in our meta-analysis, 68.8% of patients had a preserved LVEF > 40%. Thus, HBP may be advantageous over LBBP for AVB patients with preserved left ventricular function. However, due to the small number of included studies, further long-term, randomized trials are needed to explore the performance of HBP and LBBP in different pacing indications.

Compared with traditional leads with retractable screws, improvements in lead designs and delivery sheaths can increase the success rate. Barba-Pichardo et al. reported an HBP success rate of 35.4% using traditional leads (Tendril SDX electrodes, St Jude, MN, USA) in AVB patients in 2008 (29). The HBP success rate in AVB patients increased to 84% by using new tools (Select Secure, Model number 3830, Medtronic Inc., Minneapolis, MN, USA) as reported by Vijayaraman et al. (30). Moreover, with the same leads but increased experience, the success rate of HPCSP in the same center increased from 84% in 2015 (30) to 97% in 2020 (15). In our meta-analysis, six included studies used 3830 leads, and the success rate increased over time as shown in Supplementary Table 6. Due to the widespread network of left bundle branch Purkinje fibers, the capture of the left conduction system could be easily achieved and remained stable. Theoretically, LBBP may be superior to HBP in terms of a shorter procedure time and more stable pacing output (31). However, there was no difference in the procedure duration or success rate between HBP and LBBP in our study. The SUCRA results showed that HBP was similar to LBBP in procedure time (28.8% vs. 29.9%). This may be related to the learning curve of LBBP. In the future, designs of new tools and accumulating experience may increase the success rate and shorten the procedure time. Regarding the chronic evolution of the pacing output at follow-up, our analysis showed that the pacing threshold of the three pacing strategies remained stable during follow-up. The pacing impedance was significantly reduced in HBP, and a numerical pacing impedance decrease was observed in both LBBP and RVP. In the LBBP group, the R-wave amplitude increased during follow-up, whether oversensing in the long-term would occur or not remains unknown. Overall, the long-term stability of the pacing output of LBBP needs further investigation.

The risk of complications did not differ for the three pacing modalities in the meta-analysis, while SUCRA results showed that complications were least likely to occur in HBP. Supplementary Table 1 shows that for HBP, lead revision due to a progressive increase in the capture threshold accounted for 2.2% of cases (5 in 228). Other than lead revision, higher pacing thresholds with HBP may cause increased battery drainage (32), leading to a potential increase in healthcare costs, so cost-effectiveness may be another concern for HBP. New devices with longer battery life are necessary. Supplementary Table 1 shows that lead dislocation was the most common complication of LBBP (10 in 427, 2.3%), followed by septal perforation (8 in 427, 1.9%). Monitoring pacing parameters closely and assessing ventricular septal thickness by echocardiography before implantation is very important (31). With the development of new tools for precise localization and lead fixation, the risks of lead complications are expected to decline. However, further investigation of the safety of LBBP is still needed. Moreover, the mortality rate and heart failure hospitalization rate remain unknown for both procedures. Hence, large, long-term randomized controlled trials are needed to verify the efficacy, safety, and outcome of LBBP in comparison to other pacing methods.

### Study limitations

First, the sample size of the included studies was limited, which may lead to an underestimation of the actual effects, and most of the studies were observational studies, which reduced their validity compared with randomized controlled trials. Second, the difference in study design, pacing indication, follow-up time, and publication bias could cause intrinsic bias. Third, only one included study compared HBP vs. RVP, and some outcomes were indirect comparisons between HBP and RVP, leading to imbalanced network comparisons. In addition, the data from crossover design trials may influence the results. Fourth, we only included studies with AVB participants. Studies with non-selected populations or other bradycardia indications, such as sinus node disease and AV node ablation, were excluded. We also excluded the studies without the outcomes we need (33), which may cause bias. Moreover, most of the studies of physiologic pacing were performed in experienced centers, so the success rates and clinical outcomes might not apply to all clinical settings. Further multi-institutional data are needed.

## Conclusion

Our results demonstrated that HBP and LBBP were superior to RVP in paced QRS duration and preservation of LVEF for patients with atrioventricular block. LBBP was associated with a lower pacing threshold and greater R-wave amplitude than HBP. However, the stability of the pacing output of LBBP may be a concern. Further investigation of the long-term efficacy in left ventricular function and the risk of heart failure hospitalization is needed.

## Data availability statement

The original contributions presented in this study are included in the article/Supplementary material, further inquiries can be directed to the corresponding author.

## Author contributions

YZ and RD conceptualized and designed the research. YZ, YJ, and JL were responsible for the acquisition, analysis, and interpretation of data. YZ drafted the manuscript. YJ, JL, and RD critically revised the manuscript. All authors contributed to the article and approved the submitted version.

## Abbreviations

AVB: atrioventricular block
CI: confidence interval
CrI: credible interval
CRT: cardiac resynchronization therapy
HBP: His bundle pacing
HPSP: His–Purkinje system pacing
HFH: heart failure hospitalization
LBBP: left bundle branch pacing
LVEF: left ventricular ejection fraction
LVEDD: left ventricular end-diastolic diameter
MD: mean difference
NMA: network meta-analysis
PCM: pacemaker-induced cardiomyopathy
RVP: right ventricular pacing
SUCRA: surface under the cumulative ranking curve
UHF-ECG: ultra-high-frequency electrocardiography.
