# Supplementary material for: A systematic review and Bayesian network meta-analysis comparing left bundle branch pacing, his bundle branch pacing, and right ventricular pacing for atrioventricular block
Source: Front Cardiovasc Med. 2022 Oct 25;9:939850. doi: 10.3389/fcvm.2022.939850 (PMC9640391; doi:10.3389/fcvm.2022.939850)
Supplement: Supplementary file 1 [file Data_Sheet_1.docx]

***Supplemental Material***

**Electronic search strategies** ------------------------------------------------------------------------------------- **Page 3**

**Outcomes definition** ------------------------------------------------------------------------------------- **Page 3-4**

Supplemental table 1. Complications associated with pacemaker implantation.

**Risk of bias assessment** ------------------------------------------------------------------------------------- **Page 4-5**

Supplemental Table 2. Newcastle–Ottawa Scale (NOS) for the included studies

Supplemental Figure 1. ROB 2 for randomized trials.

**Results**

Supplemental Figure 2. Included studies and outcomes in the pairwise meta-analyses. --------------------- **Page 6**

Supplemental Figure 3. Included studies and outcomes in the network meta-analyses. --------------------- **Page 7**

Supplemental Figure 4-35. Forest plots for the results of pairwise meta-analysis. --------------------- **Page 7-21**

**Network plots** ---------------------------------------------------------------------------------------------------- **Page 22-23**

Supplemental Figure 36-37. Network plots for comparisons of outcomes.

**Subgroup Analysis** ---------------------------------------------------------------------------------------------- **Page 24-26**

Supplemental Table 3. Subgroup analysis by observed studies only.

**Sensitivity Analysis** ---------------------------------------------------------------------------------------------- **Page 26-30**

Supplemental Table 4. Random vs Fixed effect model for pairwise meta-analysis.

Supplemental Table 5. Bayesian vs frequentist framework for network meta-analysis.

**Publication of bias assessment** -------------------------------------------------------------------------------- **Page 30-33**

Supplemental Figure 38-40. Funnel plots and results of Egger’s test for all the outcomes.

**Discussion** --------------------------------------------------------------------------------------------------------- **Page 34**

Supplemental Table 6. Six studies use 3830 leads.

**Electronic search strategies**

1. ((His bundle pacing) OR (Hisian pacing)) AND (Atrioventricular block)
2. ((left bundle branch pacing) OR (left bundle branch area pacing)) AND (Atrioventricular block)
3. ((right ventricular pacing) OR (right ventricular myocardial pacing) OR (right ventricular apical pacing) OR (right ventricular apex pacing) OR (right ventricular septal pacing) OR (right ventricular septum pacing) OR (right ventricular outflow tract pacing)) AND (Atrioventricular block)
4. ((His bundle pacing) OR (Hisian pacing)) AND ((left bundle branch pacing) OR (left bundle branch area pacing)) AND (Atrioventricular block)

**Supplemental table 1. Complications associated with pacemaker implantation.**

| Study, Year | Complications |
| --- | --- |
| Yiran Hu 2020 | LBBP: 2 patients had lead reposition due to acute perforation of the ventricular septum during the LBBP procedure. 1 patient had lead dislodgement at follow-up.  HBP: 1 patient developed pocket hematoma and secondary infection. |
| Xiaofei Li 2021 | LBBP: 5 patients (2.1%) suffered septal perforation during the procedure without any symptoms; 1 septal perforation occurred 2 h after the procedure and resulted in dislodgement and ventricular capture failure. RVP: apical perforation occurred in 1 patient, ventricular lead dislocation occurred in 3 patients during follow-up. |
| Eriko Hasumi 2019 | There were no complications, including cardiovascular perforation, tricuspid valve injury, or loss of ventricular capture during the perioperative period or in the late phase. |
| Shigeng Zhang 2020 | NR |
| Pugazhendhi Vijayaraman 2020 | HBP: 5 patients had lead revisions due to progressive increase in capture threshold. 1 patient experienced pneumothorax requiring chest tube. 1 patient had hemorrhagic pericardial effusion and recurrent exudative pleural effusion. Pocket infection requiring explantation at 1 year occurred in 1 patient. 1 patient upgraded to BVP during follow-up. LBBP: 8 patients had loss of LB or LV septal capture due to lead dislodgements. 1 patient experienced a large hematoma requiring evacuation. 1 patient had pneumothorax. Systemic infection requiring explantation occurred in 1 patient. 2 patients upgraded to BVP during follow-up. |
| Riano Ondiviela A. 2020 | Ventricular lead dislocation, 1 in HBP, 1 in LBBP |
| Mads Brix Kronborg 2014 | 1 patient had a small pericardial effusion after the implantation and was treated conservatively. During follow-up, no patients showed signs of lead dislodgement, loss of capture, prolongation of the paced or intrinsic QRS duration, or early pacemaker infections. |

**Supplemental Table 2.** Newcastle–Ottawa Scale (NOS) for the included studies

| Study | Selection | | | | Comparability | Exposure | | | Scores |
| --- | --- | --- | --- | --- | --- | --- | --- | --- | --- |
|  | Adequate definition of cases | Representativeness of the cases | Selection of controls | Definition of controls | Control for important factor | Ascertainment of exposure | Same method of ascertainment for cases and controls | Nonresponse rate |  |
| Yiran Hu,2020 | ☆ | ☆ | ☆ | ☆ | ☆ | ☆ | ☆ | - | 7 |
| Xiaofei Li,2021 | ☆ | ☆ | ☆ | ☆ | ☆ | ☆ | ☆ | - | 7 |
| Eriko  Hasumi,2019 | - | ☆ | - | - | - | ☆ | ☆ | - | 3 |
| Shigeng  Zhang,2020 | ☆ | ☆ | ☆ | ☆ | ☆ | ☆ | ☆ | - | 7 |
| Pugazhendhi  Vijayaraman,2020 | ☆ | ☆ | ☆ | ☆ | ☆ | ☆ | ☆ | ☆ | 8 |

**Supplemental Figure 1. ROB 2 for randomized trials.**


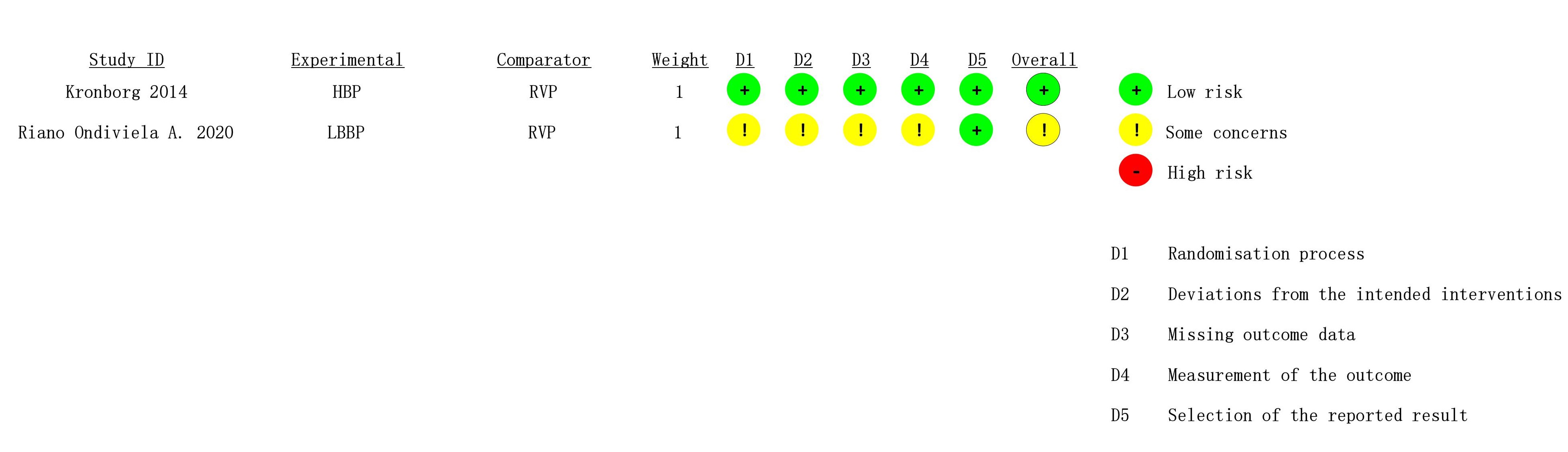


**Results**

**Supplemental Figure 2.** Included studies and outcomes in the pairwise meta-analyses.


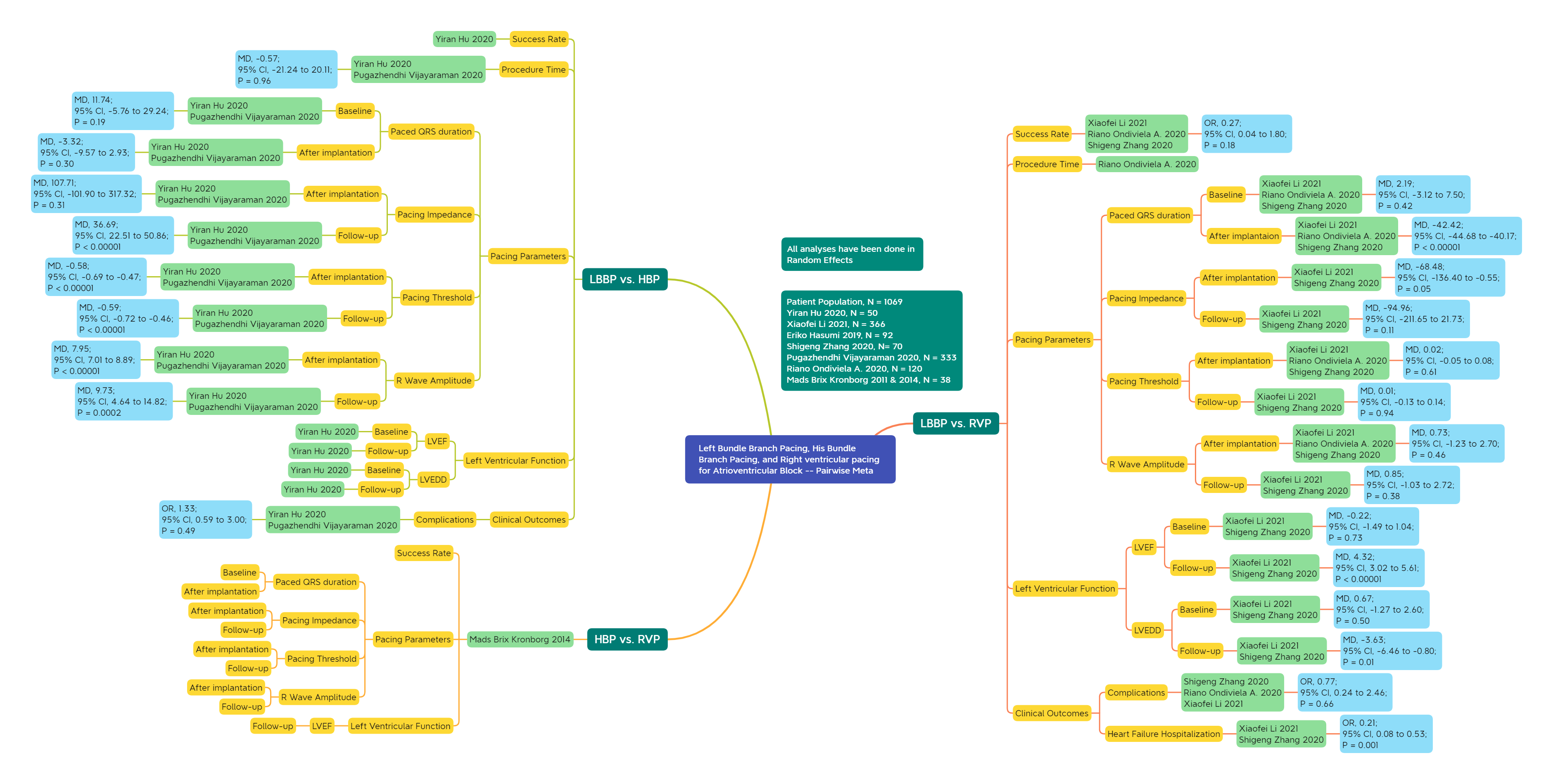


**Supplemental Figure 3.** Included studies and outcomes in the network meta-analyses. (outcomes from indirect comparisons are marked in grey.)


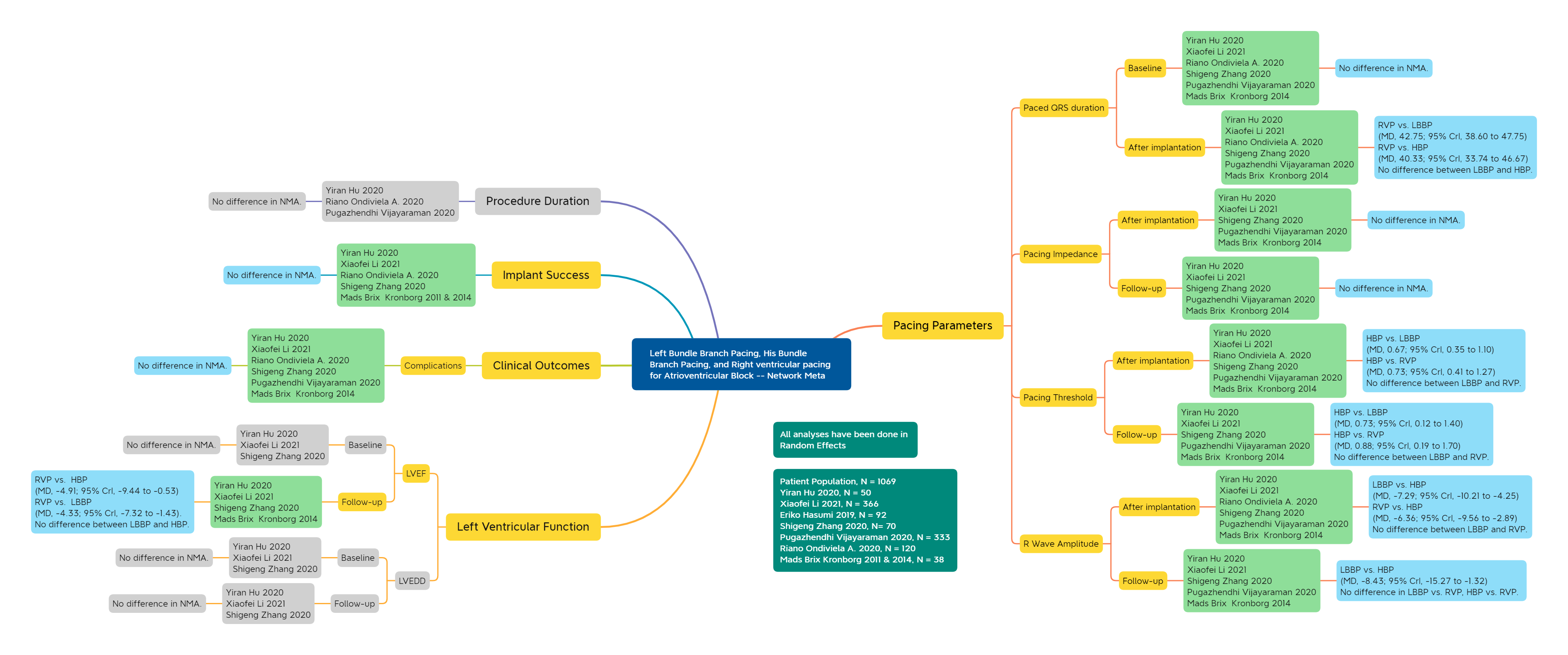


**Pairwise meta-analysis**

**Procedure Time**

**
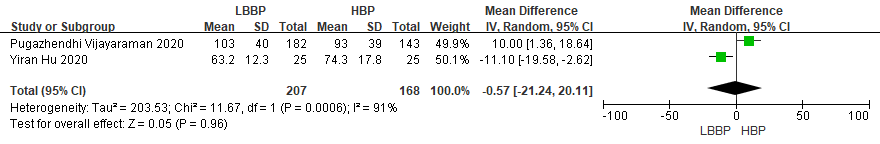
**

**Supplemental Figure 4.** There was no difference in procedure duration between LBBP and HBP.

**Implant Success Rate**

**
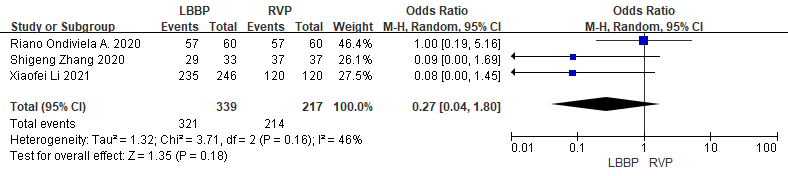
**

**Supplemental Figure 5.** There was no difference in implant success rate between LBBP and RVP.

**Pacing parameters**

**Paced QRS duration**


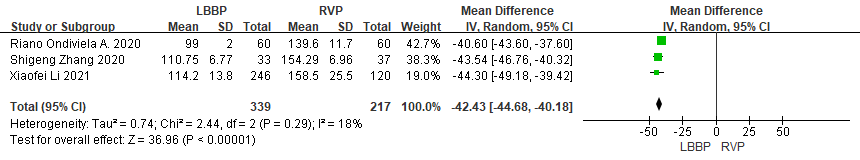


**Supplemental Figure 6.** LBBP was associated with a significantly shorter paced QRS duration than RVP.


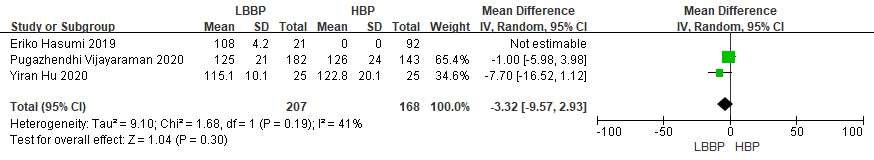


**Supplemental Figure 7.** LBBP did not shorten paced QRS duration relative to HBP.

**Pacing impedance**


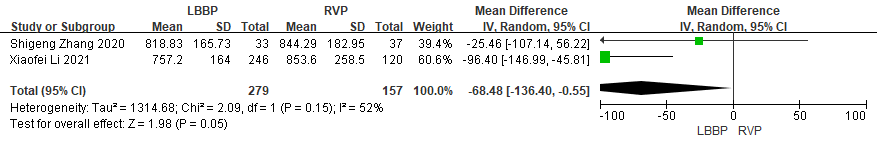


**Supplemental Figure 8.** Compared with RVP, LBBP demonstrated lower pacing impedance at the time of implantation.


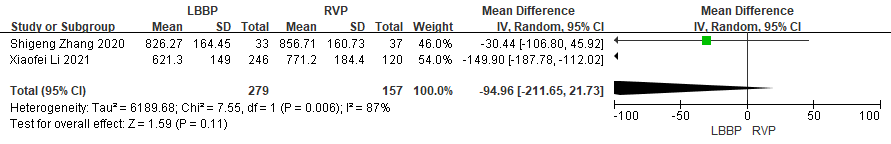


**Supplemental Figure 9.** There was no significant difference in pacing impedance at follow-up between LBBP and RVP.


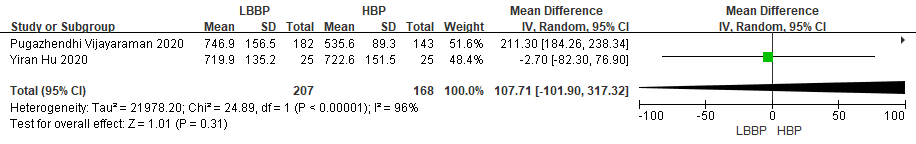


**Supplemental Figure 10.** Pacing impedance were similar between LBBP and HBP at the time of implantation.


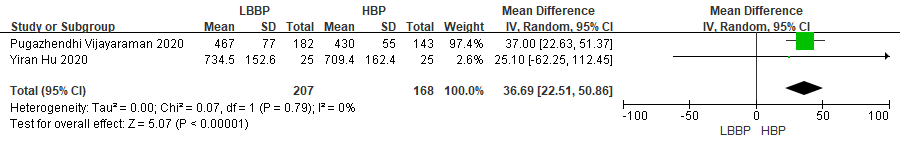


**Supplemental Figure 11.** There was a significantly higher pacing impedance in the LBBP group than in the HBP group at follow-up.

**Pacing threshold**


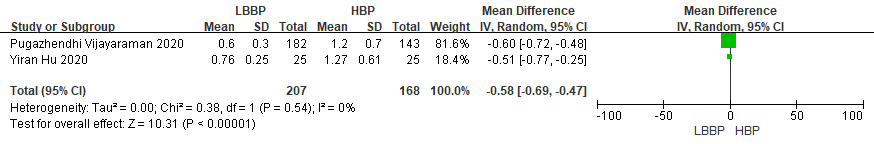


**Supplemental Figure 12.** The pacing threshold was significantly lower in the LBBP group than in the HBP group at the time of implantation


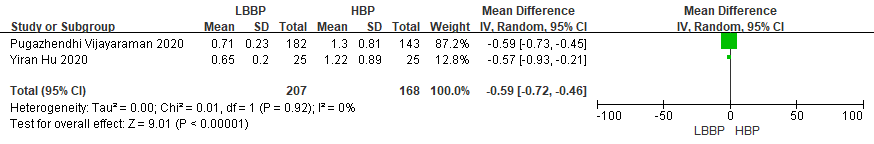


**Supplemental Figure 13.** The pacing threshold was significantly lower in the LBBP group compared with HBP at follow-up.


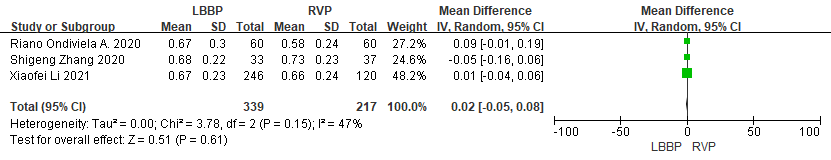


**Supplemental Figure 14.** Results for the pacing threshold did not differ between LBBP and RVP at the time of implantation.


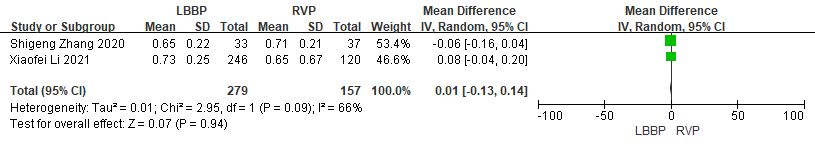


**Supplemental Figure 15.** Results for the pacing threshold did not differ between LBBP and RVP at follow-up.

**R wave amplitude**


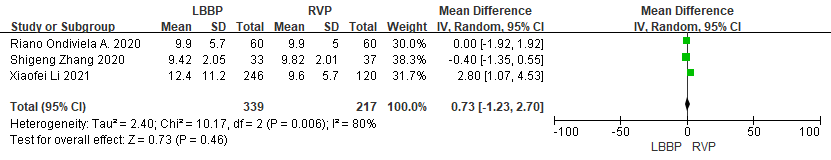


**Supplemental Figure 16.** There was no significant difference between LBBP and RVP at the time of implantation in terms of R wave amplitude.


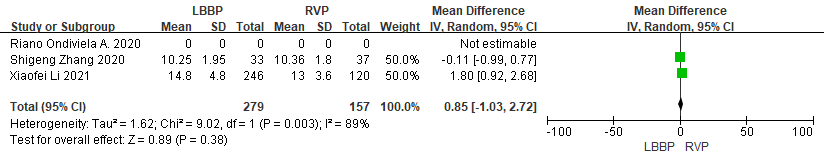


**Supplemental Figure 17.** There was no significant difference between LBBP and RVP at follow-up in terms of R wave amplitude.


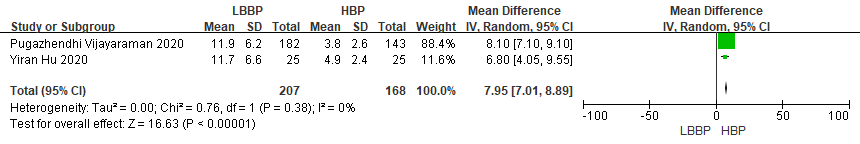


**Supplemental Figure 18.** LBBP was associated with a higher R wave amplitude in comparison with HBP at the time of implantation.


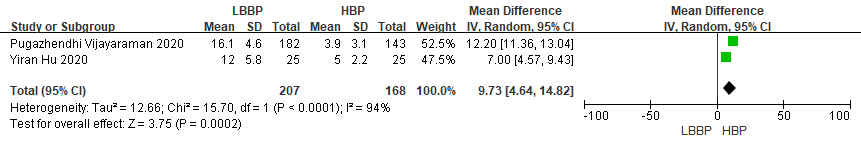


**Supplemental Figure 19.** LBBP was associated with a higher R wave amplitude than HBP at follow-up.

**Left ventricular function**

**LVEF**


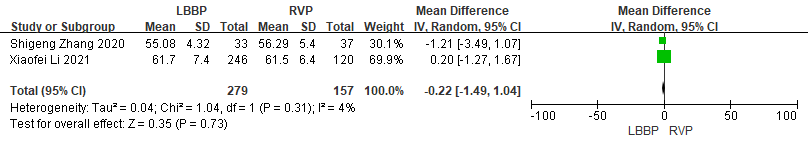


**Supplemental Figure 20.** In terms of baseline LVEF, no significant difference was found between LBBP and RVP.


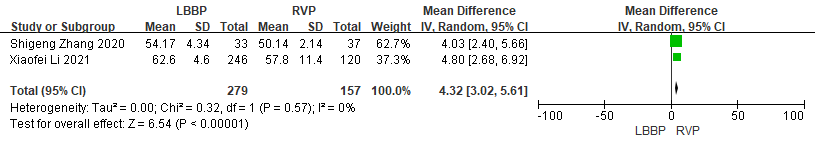


**Supplemental Figure 21.** At follow-up, LBBP demonstrated a higher LVEF than RVP.

**LVEDD**


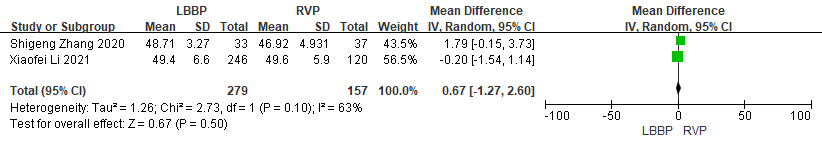


**Supplemental Figure 22.** No statistically significant difference in baseline LVEDD was found between LBBP and RVP.


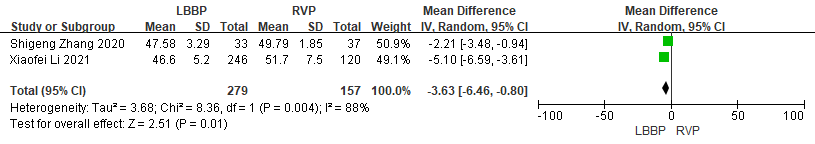


**Supplemental Figure 23.** At follow-up, LBBP was associated with a smaller LVEDD compared with RVP

**Clinical outcomes**

**Complications**

The risks of complications were similar and did not differ between LBBP and RVP, or between LBBP and HBP.


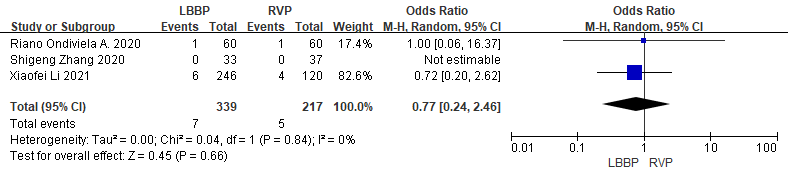


**Supplemental Figure 24.** The risks of complications were similar and did not differ between LBBP and RVP.


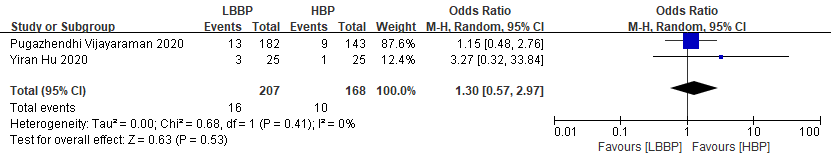


**Supplemental Figure 25.** The risks of complications were similar and did not differ between LBBP and HBP.

**Heart failure hospitalization**


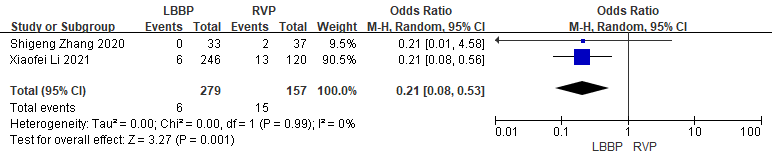


**Supplemental Figure 26.** LBBP reduced the risks of heart failure hospitalization in comparison with RVP.

**Chronic evolution of pacing parameters**

**LBBP group**


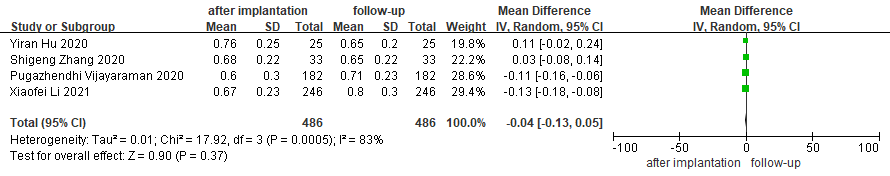


**Supplemental Figure 27.** Pacing threshold remained stable at follow-up in the LBBP group.


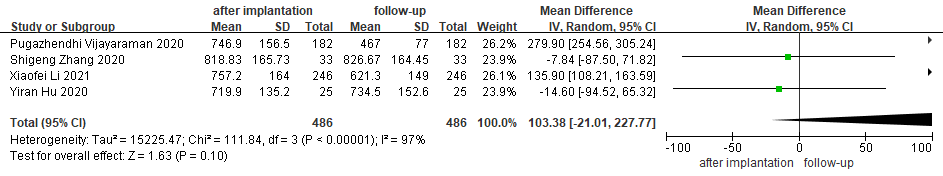


**Supplemental Figure 28.** Pacing impedance didn’t change during follow-up in the LBBP group, while there was a decreasing tendency.


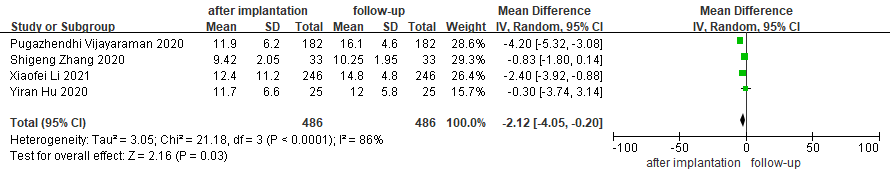


**Supplemental Figure 29.** R wave amplitude increased during follow-up in the LBBP group.

**HBP group**


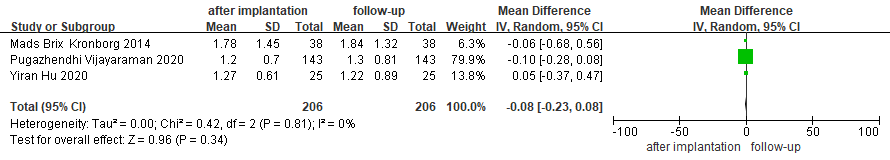


**Supplemental Figure 30.** Pacing threshold didn’t change during follow-up in the HBP group.


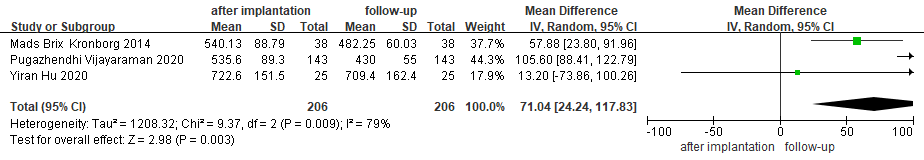


**Supplemental Figure 31.** Pacing impedance decreased at follow-up in the HBP group.


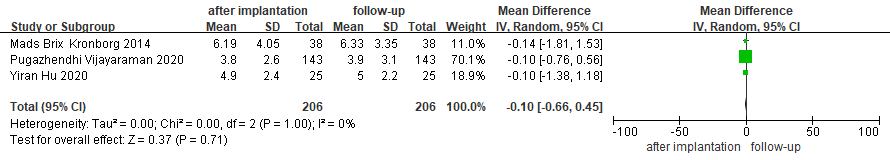


**Supplemental Figure 32.** R wave amplitude remained stable at follow-up in the HBP group.

**RVP group**

**
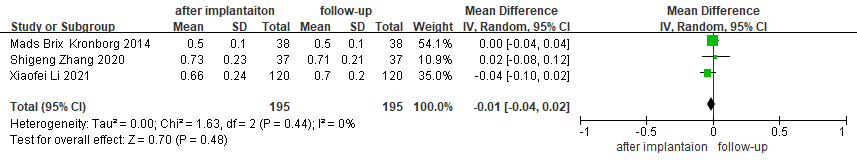
**

**Supplemental Figure 33.** Pacing threshold remained stable at follow-up in the RVP group.


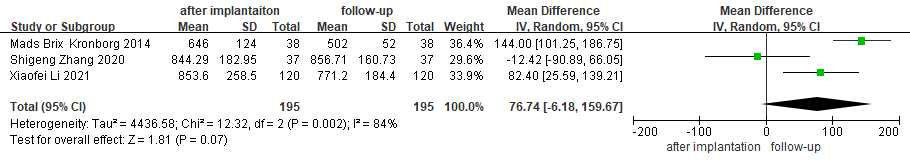


**Supplemental Figure 34.** Pacing impedance didn’t change at follow-up in the RVP group, but there was a decreasing tendency at follow-up.


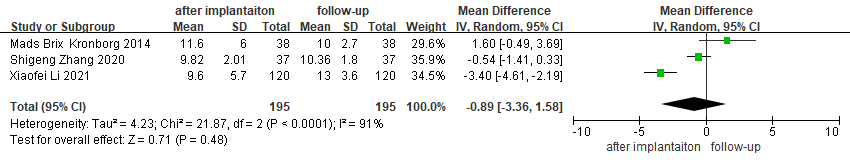


**Supplemental Figure 35.** R wave amplitude didn’t change at follow-up in the RVP group.

**Supplemental Figure 36 & 37.** Network plots for comparisons of outcomes.


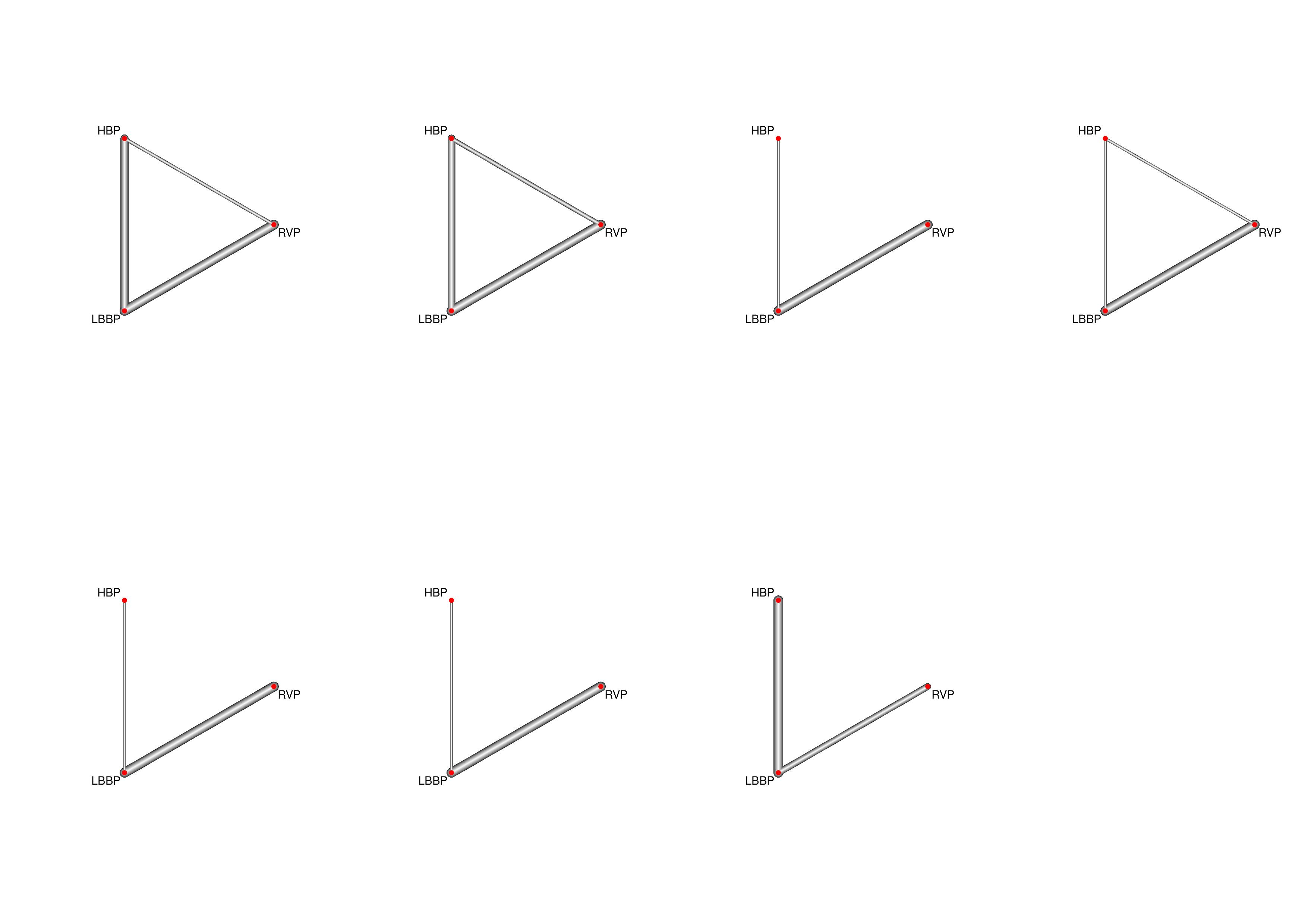


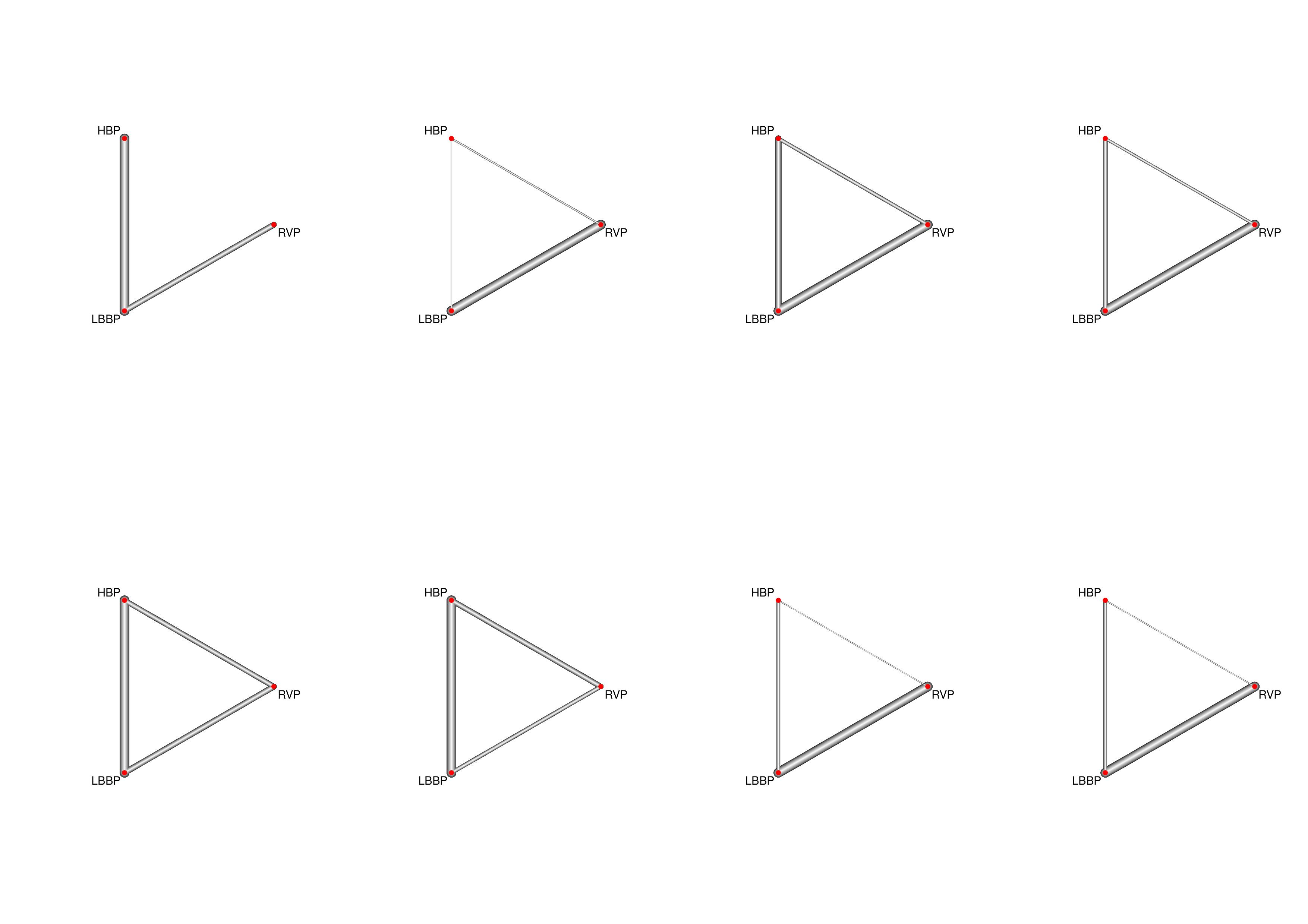


1. Procedure time

B. Success rate

C. Baseline QRS duration

D. Paced QRS duration

E. Pacing impedance after implantation

F. Pacing impedance at follow-up

G. Pacing threshold after implantation

H. Pacing threshold at follow-up

I. R wave amplitudes after implantation

J. R wave amplitudes at follow-up

K. Baseline LVEF

L. LVEF at follow-up

M. Baseline LVEDD

N. LVEDD at follow-up

O. Complications

**Subgroup Analysis**

**Supplemental Table 3.** Subgroup analysis by observed studies only. (Data are effect size (odds ratio or mean difference) and 95% confidence interval (CI). Different results are marked in grey.)

| Outcome | Comparison | Main analysis (observed studies and RCTs) | Subgroup analysis (observed studies only) |  |
| --- | --- | --- | --- | --- |
|  |  |  |  |  |
| Procedure duration (min) | LBBP vs. HBP | -0.63(-24.64,23.54) | NA |  |
|  | LBBP vs. RVP | 24.26(-10.48,59.21) |  |  |
|  | HBP vs. RVP | 24.86(-17.93,66.58) |  |  |
| Success rate | LBBP vs. HBP | 0.56(-2.54,3.36) | 0.85[-2.48,4.47] |  |
|  | LBBP vs. RVP | -2.06(-5.04, -0.04) | **-20.75[-84.20,-3.45]** |  |
|  | HBP vs. RVP | -2.65(-5.90,0.20) | **-21.35[-84.54,-3.86]** |  |
| Baseline QRS duration (ms) | LBBP vs. HBP | 10.12(-4.30,21.92) | 13.89[-6.38,28.24] |  |
|  | LBBP vs. RVP | 3.86(-6.61,14.38) | 1.96[-13.50,17.14] |  |
|  | HBP vs. RVP | -6.19(-19.15,9.23) | -12.00[-32.18,13.86] |  |
| Paced QRS duration (ms) | LBBP vs. HBP | -2.45(-8.84,3.16) | -3.23[-21.02,13.51] |  |
|  | LBBP vs. RVP | **-42.75(-47.75, -38.60)** | **-43.75[-60.52,-27.13]** |  |
|  | HBP vs. RVP | **-40.33(-46.67, -33.74)** | **-40.59[-64.03,-15.99]** |  |
| Baseline  pacing impedance (Ω) | LBBP vs. HBP | 95.56(-68.86,253.21) | 112.85[-96.05,311.59] |  |
|  | LBBP vs. RVP | -139.22(-323.81,48.50) | -63.04[-264.29,141.63] |  |
|  | HBP vs. RVP | -44.05(-204.39,118.86) | -175.21[-452.84,116.39] |  |
| Follow-up  pacing impedance (Ω) | LBBP vs. HBP | 2.89(-112.49,121.58) | 33.73[-92.71,163.71] |  |
|  | LBBP vs. RVP | -67.35(-183.21,49.64) | -101.83[-222.35,38.73] |  |
|  | HBP vs. RVP | -70.57(-203.51,62.04) | -136.33[-310.72,55.24] |  |
| Baseline  pacing threshold (V) | LBBP vs. HBP | **-0.67(-1.10,-0.35)** | **-0.58[-0.86,-0.26]** |  |
|  | LBBP vs. RVP | 0.06(-0.18,0.42) | -0.01[-0.32,0.26] |  |
|  | HBP vs. RVP | **0.73(0.41,1.27)** | **0.58[0.12,0.95]** |  |
| Follow-up  pacing threshold (V) | LBBP vs. HBP | **-0.73(-1.40,-0.12)** | **-0.57[-0.84,-0.27]** |  |
|  | LBBP vs. RVP | 0.14(-0.45,0.82) | -0.01[-0.29,0.24] |  |
|  | HBP vs. RVP | **0.88(0.19,1.70)** | **0.56[0.14,0.93]** |  |
| Baseline  R wave amplitude (mV) | LBBP vs. HBP | **7.29(4.25,10.21)** | **7.59[1.81,13.20]** |  |
|  | LBBP vs. RVP | 0.92(-1.55,3.48) | 1.03[-4.39,6.69] |  |
|  | HBP vs. RVP | **-6.36(-9.56, -2.89)** | -6.56[-14.21,1.55] |  |
| Follow-up  R wave amplitude (mV) | LBBP vs. HBP | **8.43(1.32,15.27)** | **9.87[1.10,18.43]** |  |
|  | LBBP vs. RVP | 2.13(-4.84,9.15) | 0.85[-7.56,9.21] |  |
|  | HBP vs. RVP | -6.28(-14.21,1.76) | -9.06[-20.97,3.06] |  |
| Baseline  LVEF* (%) | LBBP vs. HBP | -1.03(-6.26,4.75) | NA |  |
|  | LBBP vs. RVP | -0.31(-2.22,1.46) |  |  |
|  | HBP vs. RVP | 0.66(-5.32,6.34) |  |  |
| Follow-up  LVEF (%) | LBBP vs. HBP | -0.57(-5.16,3.96) | -0.80[-7.81,5.80] |  |
|  | LBBP vs. RVP | **4.33(1.43,7.32)** | **4.36[0.79,8.02]** |  |
|  | HBP vs. RVP | **4.91(0.53,9.44)** | 5.14[-2.30,13.02] |  |
| Baseline  LVEDD* (mm) | LBBP vs. HBP | -0.55(-5.58,4.37) | NA |  |
|  | LBBP vs. RVP | 0.57(-1.30,2.59) |  |  |
|  | HBP vs. RVP | 1.13(-4.17,6.57) |  |  |
| Follow-up  LVEDD (mm) | LBBP vs. HBP | -0.19(-7.03,6.87) | NA |  |
|  | LBBP vs. RVP | -3.61(-8.21,0.88) |  |  |
|  | HBP vs. RVP | -3.45(-11.72,4.65) |  |  |
| Complications | LBBP vs. HBP | 0.36(-0.77,1.64) | 0.38[-0.76,1.67] |  |
|  | LBBP vs. RVP | -0.18(-1.56,1.30) | -0.21[-1.97,1.59] |  |
|  | HBP vs. RVP | -0.55(-2.35,1.29) | -0.59[-2.79,1.59] |  |

**Sensitivity Analysis**

**Supplemental Table 4.** Random vs Fixed effect model for pairwise meta-analysis. (Data are effect size (odds ratio or mean difference) and 95% confidence interval (CI). Different results are marked in grey.)

| Outcome | Comparison | Main analysis (Random effects model) | Sensitivity analysis (Fixed effects model) |  |
| --- | --- | --- | --- | --- |
|  |  |  |  |  |
| Procedure duration | LBBP vs. HBP | -0.57 [-21.24, 20.11] | -0.57 [-21.24, 20.11] |  |
|  | LBBP vs. RVP | NA | NA |  |
| Success rate | LBBP vs. HBP | NA | NA |  |
|  | LBBP vs. RVP | 0.27 [0.04,1.80] | **0.26 [0.08, 0.80]** |  |
| Baseline QRS duration | LBBP vs. HBP | 11.74 [-5.76, 29.24] | **16.63 [10.55, 22.71]** |  |
|  | LBBP vs. RVP | 2.20 [-3.11, 7.51] | 2.22 [-1.62, 6.06] |  |
| Paced QRS duration | LBBP vs. HBP | -3.32 [-9.57, 2.93] | -2.62 [-6.95, 1.72] |  |
|  | LBBP vs. RVP | **-42.43 [-44.68, -40.18]** | **-42.36 [-44.36, -40.35]** |  |
| Baseline  pacing impedance | LBBP vs. HBP | 107.71 [-101.90, 317.32] | **189.16 [163.55, 214.76]** |  |
|  | LBBP vs. RVP | -68.48 [-136.40, -0.55] | -76.73 [-119.74, -33.73] |  |
| Follow-up  pacing impedance | LBBP vs. HBP | **36.69 [22.51, 50.86]** | **36.69 [22.51, 50.86]** |  |
|  | LBBP vs. RVP | -94.96 [-211.65, 21.73] | **-126.30 [-160.24, -92.37]** |  |
| Baseline  pacing threshold | LBBP vs. HBP | **-0.58 [-0.69, -0.47]** | **-0.58 [-0.69, -0.47]** |  |
|  | LBBP vs. RVP | 0.02 [-0.05, 0.08] | 0.02 [-0.03, 0.06] |  |
| Follow-up  pacing threshold | LBBP vs. HBP | **-0.59 [-0.72, -0.46]** | **-0.59 [-0.72, -0.46]** |  |
|  | LBBP vs. RVP | 0.01 [-0.13, 0.14] | -0.00 [-0.08, 0.07] |  |
| Baseline  R wave amplitude | LBBP vs. HBP | **7.95 [7.01, 8.89]** | **7.95 [7.01, 8.89]** |  |
|  | LBBP vs. RVP | 0.73 [-1.23, 2.70] | 0.29 [-0.48, 1.06] |  |
| Follow-up  R wave amplitude | LBBP vs. HBP | **9.73 [4.64, 14.82]** | **11.65 [10.85, 12.44]** |  |
|  | LBBP vs. RVP | 0.85 [-1.03, 2.72] | **0.85 [0.22, 1.47]** |  |
| Baseline  LVEF* | LBBP vs. HBP | NA | NA |  |
|  | LBBP vs. RVP | -0.22 [-1.49, 1.04] | -0.21 [-1.45, 1.02] |  |
| Follow-up  LVEF | LBBP vs. HBP | NA | NA |  |
|  | LBBP vs. RVP | **4.32 [3.02,5.61]** | **4.32 [3.02, 5.61]** |  |
| Baseline  LVEDD* | LBBP vs. HBP | NA | NA |  |
|  | LBBP vs. RVP | 0.67 [-1.27, 2.60] | 0.44 [-0.66, 1.54] |  |
| Follow-up  LVEDD | LBBP vs. HBP | NA | NA |  |
|  | LBBP vs. RVP | **-3.63 [-6.46, -0.80]** | **-3.43 [-4.39, -2.46]** |  |
| Complications | LBBP vs. HBP | 1.30 [0.57, 2.97] | 1.30 [0.57, 2.97] |  |
|  | LBBP vs. RVP | 0.77 [0.24, 2.46] | 0.77 [0.24, 2.48] |  |
| Heart-failure hospitalization | LBBP vs. HBP | NA | NA |  |
|  | LBBP vs. RVP | **0.21 [0.08, 0.53]** | **0.21 [0.08, 0.53]** |  |
| Chronic evolution of pacing parameters | | | |  |
| Pacing threshold | LBBP | -0.04 [-0.13, 0.05] | **-0.09 [-0.12, -0.06]** |  |
|  | HBP | -0.08 [-0.23, 0.08] | -0.08 [-0.23, 0.08] |  |
|  | RVP | -0.01 [-0.04, 0.02] | -0.01 [-0.04, 0.02] |  |
| Pacing impedance | LBBP | 103.38 [-21.01, 227.77] | **191.96 [174.22, 209.71]** |  |
|  | HBP | **71.04 [24.24, 117.83]** | **93.43 [78.31, 108.54]** |  |
|  | RVP | 76.74 [-6.18, 159.67] | **100.36 [69.03, 131.68]** |  |
| R wave amplitude | LBBP | **-2.12 [-4.05, -0.20]** | **-2.22 [-2.87, -1.57]** |  |
|  | HBP | -0.10 [-0.66, 0.45] | -0.10 [-0.66, 0.45] |  |
|  | RVP | -0.89 [-3.36, 1.58] | **-1.20 [-1.87, -0.53]** |  |

**Supplemental Table 5.** Bayesian vs frequentist framework for network meta-analysis. (Data are effect size (odds ratio or mean difference) and 95% confidence interval (CI). Different results are marked in grey.)

| Outcome | Comparison | Main analysis (Bayesian framework) | Sensitivity analysis (Frequentist framework) |  |
| --- | --- | --- | --- | --- |
|  |  |  |  |  |
| Procedure duration (min) | LBBP vs. HBP | -0.63(-24.64,23.54) | -0.74[-6.80,5.30] |  |
|  | LBBP vs. RVP | 24.26(-10.48,59.21) | 24.60[-5.26,54.46] |  |
|  | HBP vs. RVP | 24.86(-17.93,66.58) | **25.34[13.25,37.44]** |  |
| Success rate | LBBP vs. HBP | 0.56(-2.54,3.36) | 1.12[0.98,1.27] |  |
|  | LBBP vs. RVP | -2.06(-5.04, -0.04) | **-1.05[-1.07,-1.02]** |  |
|  | HBP vs. RVP | -2.65(-5.90,0.20) | **-1.16[-1.33,-1.03]** |  |
| Baseline QRS duration (ms) | LBBP vs. HBP | 10.12(-4.30,21.92) | **10.01[0.37,19.65]** |  |
|  | LBBP vs. RVP | 3.86(-6.61,14.38) | **4.00[3.23,11.24]** |  |
|  | HBP vs. RVP | -6.19(-19.15,9.23) | -6.00[-16.36,4.35] |  |
| Paced QRS duration (ms) | LBBP vs. HBP | -2.45(-8.84,3.16) | -2.10[-5.95,1.76] |  |
|  | LBBP vs. RVP | **-42.75(-47.75, -38.60)** | **-42.53[-44.66,-40.44]** |  |
|  | HBP vs. RVP | **-40.33(-46.67, -33.74)** | **-40.45[-44.56,-36.35]** |  |
| Baseline  pacing impedance (Ω) | LBBP vs. HBP | 95.56(-68.86,253.21) | 93.70[-40.02,227.43] |  |
|  | LBBP vs. RVP | -139.22(-323.81,48.50) | -45.16[-179.94,89.63] |  |
|  | HBP vs. RVP | -44.05(-204.39,118.86) | -138.86[-293.01,15.30] |  |
| Follow-up  pacing impedance (Ω) | LBBP vs. HBP | 2.89(-112.49,121.58) | 3.70[-96.56,103.96] |  |
|  | LBBP vs. RVP | -67.35(-183.21,49.64) | -66.58[-166.88,33.72] |  |
|  | HBP vs. RVP | -70.57(-203.51,62.04) | -70.29[-183.94,43.36] |  |
| Baseline  pacing threshold (V) | LBBP vs. HBP | **-0.67(-1.10,-0.35)** | **-0.62[-0.73,-0.51]** |  |
|  | LBBP vs. RVP | 0.06(-0.18,0.42) | 0.04[-0.06,0.15] |  |
|  | HBP vs. RVP | **0.73(0.41,1.27)** | **0.64[0.52,0.76]** |  |
| Follow-up  pacing threshold (V) | LBBP vs. HBP | **-0.73(-1.40,-0.12)** | **-0.72[-0.95,-0.49]** |  |
|  | LBBP vs. RVP | 0.14(-0.45,0.82) | 0.11[-0.08,0.30] |  |
|  | HBP vs. RVP | **0.88(0.19,1.70)** | **0.82[0.55,1.10]** |  |
| Baseline  R wave amplitude (mV) | LBBP vs. HBP | **7.29(4.25,10.21)** | **7.26[5.32,9.20]** |  |
|  | LBBP vs. RVP | 0.92(-1.55,3.48) | 0.94[-0.66,2.55] |  |
|  | HBP vs. RVP | **-6.36(-9.56, -2.89)** | **-6.32[-8.52,-4.13]** |  |
| Follow-up  R wave amplitude (mV) | LBBP vs. HBP | **8.43(1.32,15.27)** | **8.42[4.57,12.26]** |  |
|  | LBBP vs. RVP | 2.13(-4.84,9.15) | 2.11[-1.65,5.88] |  |
|  | HBP vs. RVP | -6.28(-14.21,1.76) | **-6.30[-10.69,-1.90]** |  |
| Baseline  LVEF* (%) | LBBP vs. HBP | -1.03(-6.26,4.75) | -1.60[-6.99,3.79] |  |
|  | LBBP vs. RVP | -0.31(-2.22,1.46) | -0.23[-1.49,1.04] |  |
|  | HBP vs. RVP | 0.66(-5.32,6.34) | 1.37[-4.17,6.92] |  |
| Follow-up  LVEF (%) | LBBP vs. HBP | -0.57(-5.16,3.96) | -0.79[-4.25,2.66] |  |
|  | LBBP vs. RVP | **4.33(1.43,7.32)** | **4.31[3.03,5.58]** |  |
|  | HBP vs. RVP | **4.91(0.53,9.44)** | **5.10[1.65,8.55]** |  |
| Baseline  LVEDD* (mm) | LBBP vs. HBP | -0.55(-5.58,4.37) | -0.80[-5.30,3.70] |  |
|  | LBBP vs. RVP | 0.57(-1.30,2.59) | 0.44[-0.66,1.54] |  |
|  | HBP vs. RVP | 1.13(-4.17,6.57) | 1.24[-3.39,5.87] |  |
| Follow-up  LVEDD (mm) | LBBP vs. HBP | -0.19(-7.03,6.87) | -0.20[-5.50,5.10] |  |
|  | LBBP vs. RVP | -3.61(-8.21,0.88) | **-3.62[-6.46,-0.80]** |  |
|  | HBP vs. RVP | -3.45(-11.72,4.65) | -3.43[-9.44,2.58] |  |
| Complications | LBBP vs. HBP | 0.36(-0.77,1.64) | 1.28 [0.59; 2.76] |  |
|  | LBBP vs. RVP | -0.18(-1.56,1.30) | -1.30[-4.03,-0.42] |  |
|  | HBP vs. RVP | -0.55(-2.35,1.29) | -1.66[-6.53,-0.42] |  |

**Supplemental Figure 38 & 39 & 40. Funnel plots for all the outcomes.**

1. Procedure duration, p = 0.9423 (Egger). B. Success rate, p = 0.8475 (Egger).
2. Baseline QRS duration, p = 0.3361 (Egger). D. Paced QRS duration, p = 0.3295 (Egger).

E. Pacing impedance after implantation, p = 0.0860 (Egger). F. Pacing impedance at follow-up, p = 0.8476 (Egger).

G. Pacing threshold after implantation, p = 0.8369 (Egger). H. Pacing threshold at follow-up, p = 0.4206 (Egger).

1. R wave amplitudes after implantation, p = 0.7277 (Egger). J. R wave amplitudes at follow-up, p = 0.3692 (Egger).

K. Baseline LVEF, p = 0.7296 (Egger). L. LVEF at follow-up, p = 0.7843 (Egger).

M. Baseline LVEDD, p = 0.7221 (Egger). N. LVEDD at follow-up, p = 0.8626 (Egger).

O. Complications, p = 0.1940 (Egger).


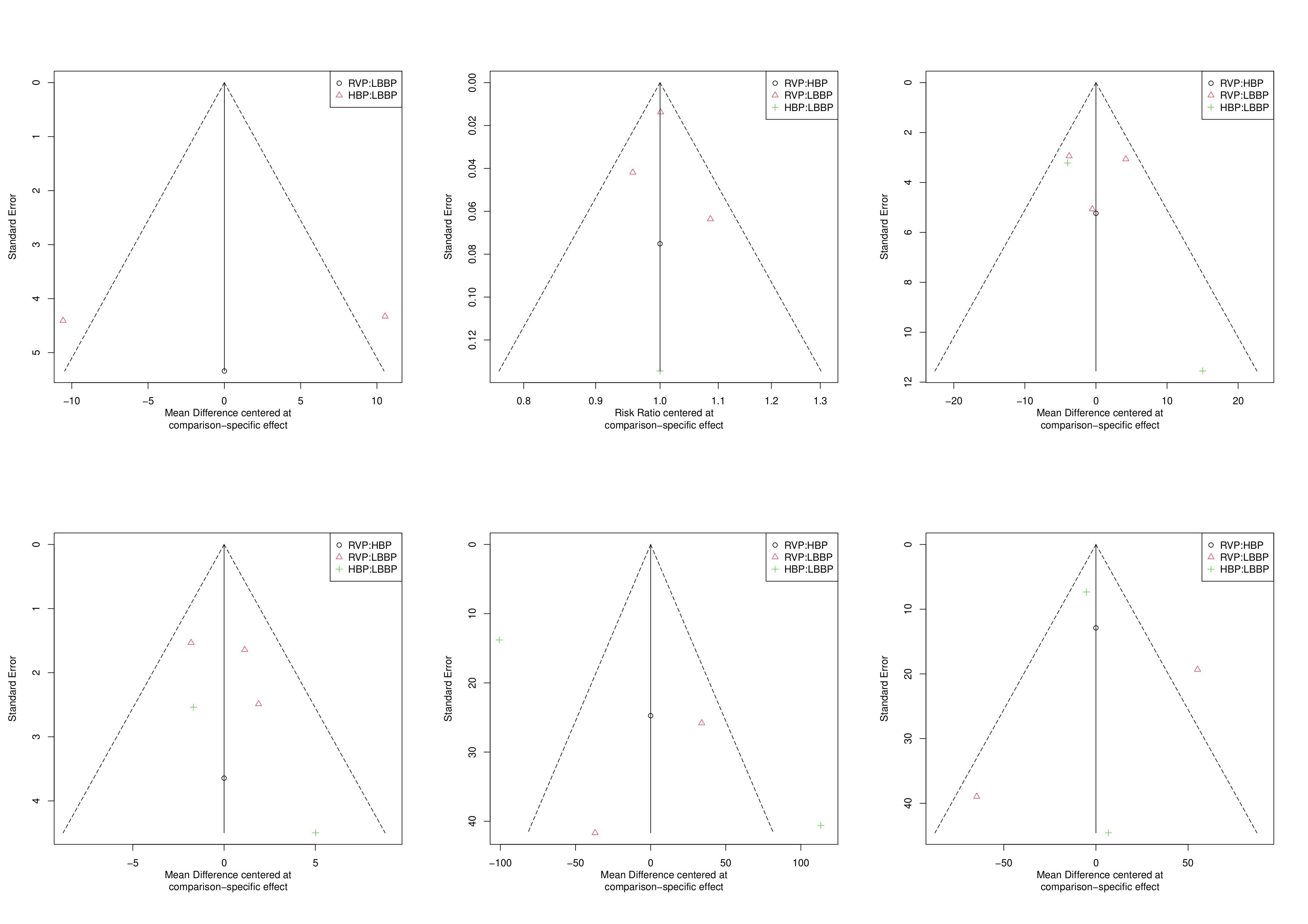


**A**

**F**

**E**

**D**

**C**

**B**


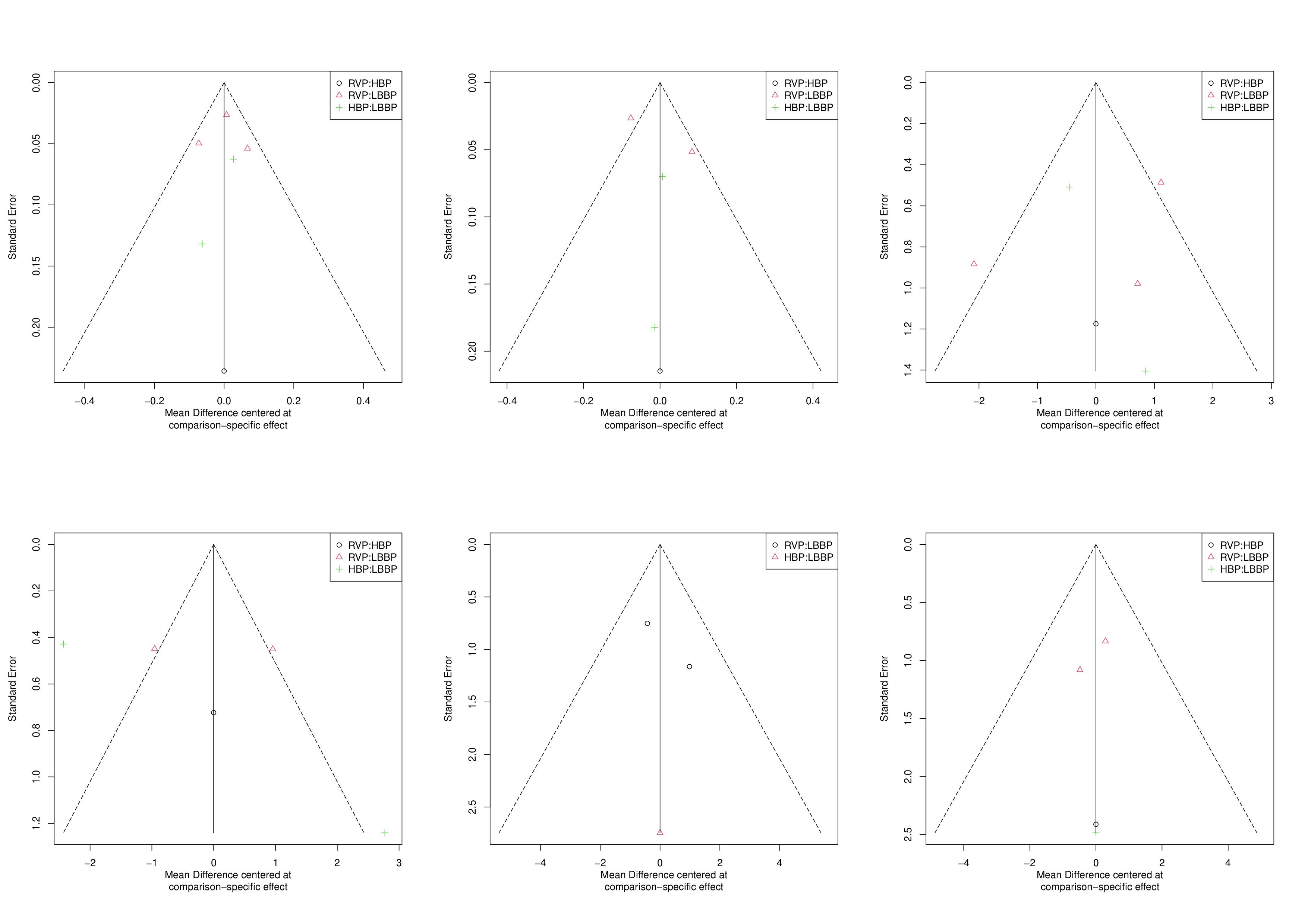


**G**

**L**

**K**

**J**

**I**

**H**


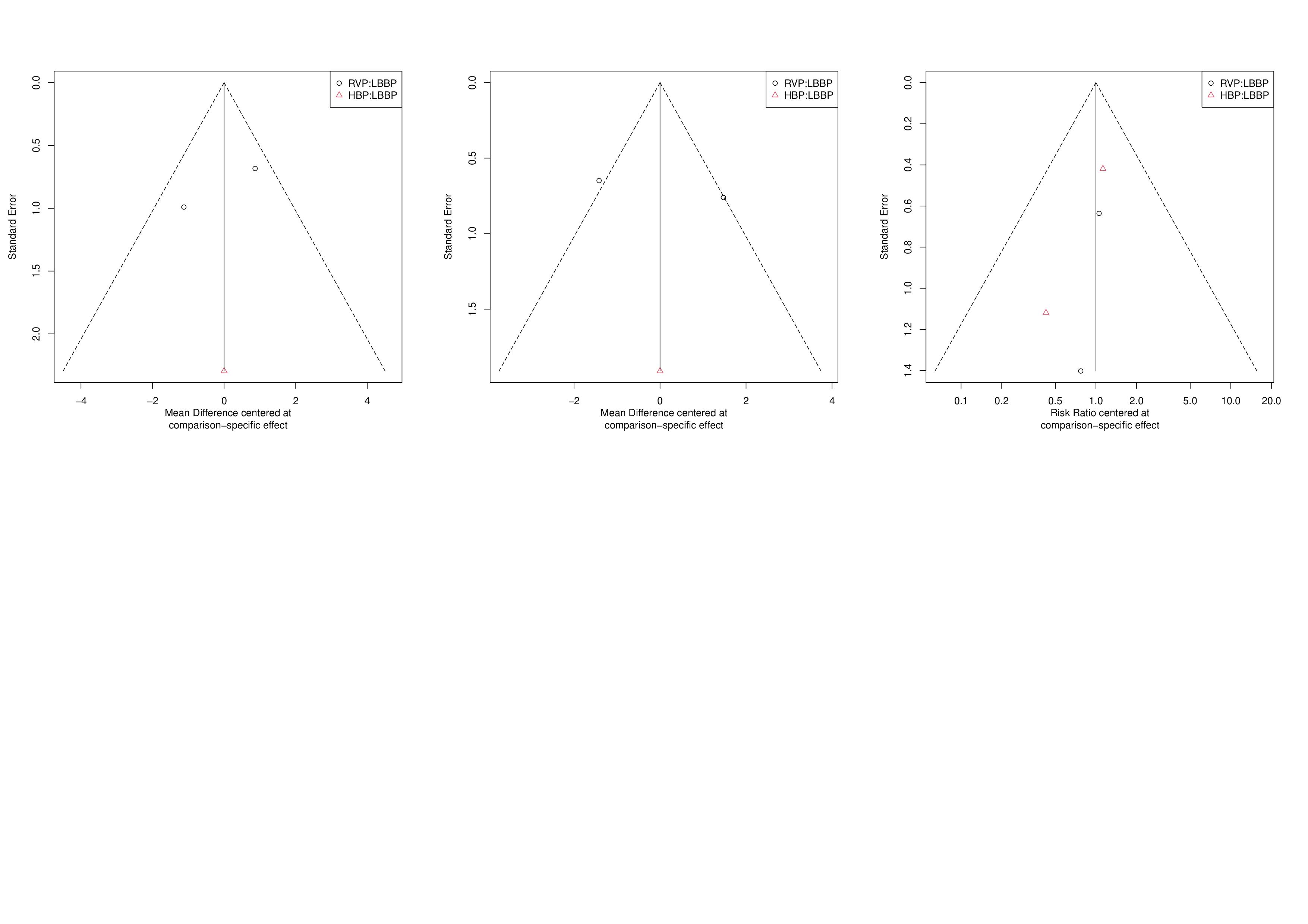


**M**

**O**

**N**

**Discussion**

**Supplemental Table 6.** Six studies use 3830 leads.

| **Study** | **Year** | **Success Rate** | **Pacing lead** | **Delivery sheath** |
| --- | --- | --- | --- | --- |
| Mads Brix Kronborg 2014 | 2007.09~2009.12 | HBP: 84% | HBP: Select Secure 3830 lead | C304 sheath |
| Shigeng Zhang 2020 | 2018.01~2018.12 | LBBP: 87.9% | LBBP: Select Secure 3830 lead | C315 sheath |
| Eriko Hasumi 2019 | ~2019 | LBBP: 81% HBP: 64% | LBBP: Select Secure 3830 lead | C315 sheath |
| Pugazhendhi Vijayaraman 2020 | 2018.04~2020.10 | NR | LBBP & HBP: Select Secure 3830 lead | C315 or C304 sheath |
| Yiran Hu 2020 | 2018.08~2019.04 | LBBP: 88% HBP: 76% | LBBP & HBP: Select Secure 3830 lead | C315 sheath |
| Riano Ondiviela A. 2020 | 2020.05~2020.10 | LBBP:95% | NR | NR |
| Xiaofei Li 2021 | 2019-2021 | LBBP: 95.5% | LBBP: Select Secure 3830 lead | C315 sheath |
